# Anthropogenic bottom‐up and top‐down impacts on boreal breeding waterbirds

**DOI:** 10.1002/ece3.11136

**Published:** 2024-03-10

**Authors:** Sari Holopainen, Kim Jaatinen, Toni Laaksonen, Andreas Lindén, Petri Nummi, Markus Piha, Hannu Pöysä, Tero Toivanen, Veli‐Matti Väänänen, Mikko Alhainen, Aleksi Lehikoinen

**Affiliations:** ^1^ Luonnontieteellinen Keskusmuseo, Finnish Museum of Natural History University of Helsinki Helsinki Finland; ^2^ Nature and Game Management Trust Finland Degerby Finland; ^3^ Department of Biology University of Turku Turku Finland; ^4^ Natural Resources Institute Finland Luke Helsinki Finland; ^5^ Department of Forest Sciences University of Helsinki Helsinki Finland; ^6^ Natural Resources Institute Finland Luke Joensuu Finland; ^7^ Department of Environmental and Biological Sciences University of Eastern Finland Joensuu Finland; ^8^ BirdLife Finland Helsinki Finland; ^9^ Finnish Wildlife Agency Helsinki Finland; ^10^ Present address: Department of Forest Sciences University of Helsinki Helsinki Finland

**Keywords:** alien predators, brownification, eutrophication, pH, waterfowl, wetland

## Abstract

Wetland habitats are changing under multiple anthropogenic pressures. Nutrient leakage and pollution modify physico‐chemical state of wetlands and affect the ecosystem through bottom‐up processes, while alien predators affect the ecosystems in a top‐down manner. Boreal wetlands are important breeding areas for several waterbird species, the abundances of which potentially reflect both bottom‐up and top‐down ecosystem processes. Here, we use long‐term national monitoring data gathered from c. 130 waterbird breeding sites in Finland from the 1980s to the 2020s. We hypothesised that the physico‐chemical state of the waters and increasing alien predator abundance both play a role in steering the waterbird population trends. We set out to test this hypothesis by relating population changes of 17 waterbird species to changes in water chemistry and to regional alien predator indices while allowing species‐specific effects to vary with foraging niche (dabblers, invertivore divers, piscivorous divers, herbivores), nesting site, female mass and habitat (oligotrophic, eutrophic). We found niche and nesting site‐specific, habitat‐dependent changes in waterbird numbers. While the associations with higher phosphorus levels and browning water were in overall positive at the oligotrophic lakes, the numbers of invertivore and piscivore diving ducks were most strongly negatively associated with higher phosphorus levels and browning water at the eutrophic lakes. Furthermore, increased pH levels benefitted piscivores. Invertivore diving duck species nesting on the wetlands had declined most on sites with high alien predator indices. Large herbivorous species and species preferring oligotrophic lakes seem to be successful. We conclude that the large‐scale breeding waterbird decline in Finland is closely connected to both bottom‐up and top‐down processes, where negative associations are emphasised especially at eutrophic lakes. Niche‐, nest site‐ and habitat‐specific management actions are required to conserve declining waterbird populations. Managing wetlands on catchments level together with alien predator control may provide important approaches to future wetland management.

## Graphical abstract

Waterbird abundances reflect both bottom‐up and top‐down processes. Changes in water quality and alien predators have had asymmetric long‐term associations with waterbirds. Waterbird foraging strategy and nesting site affect the sensitivity to the changes, but the effects depend also on natural trophic status of the lakes.

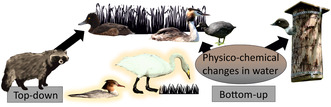

## 1 INTRODUCTION

Wetlands worldwide are modified and affected by multiple anthropogenic stressors including both large‐scale (e.g. acid precipitation, climate change) and local (e.g. eutrophication, invasive alien species) effects (Bradshaw et al., 2009; de Wit et al., 2016; Holopainen et al., 2021; Holopainen & Lehikoinen, 2022; Mischenko et al., 2020; Schindler, 1998; Weyhenmeyer et al., 2014). Anthropogenic pressures can have strong impacts on the biodiversity of wetlands, influencing all trophic levels from primary producers to higher level consumers (Christensen et al., 2006; Nilsson, 1978).

The main driver of ecosystem stress in most wetlands is eutrophication, caused by diffuse nutrient loading by macronutrients such as nitrogen and phosphorus (Birk et al., 2020; Smith, 2003). The increased nutrient and sediment flows can have strong effects on biodiversity in wetlands. This is because physico‐chemical quality elements, such as light, acidification, and nutrient conditions, are expected to support the biological quality elements, such as phytoplankton, benthic invertebrates and macrophytes, of the wetlands (Kristensen et al., 2018). Bottom‐up effects are versatile: for example, brownification (a result of increased levels of DOC/TOC) of the water together with decreasing water clarity in lakes affects the light conditions and productivity of lakes (Blanchet et al., 2022; Karlsson et al., 2009; Seekell et al., 2015) and may be accompanied by a progressive loss of aquatic macrophytes (Choudhury et al., 2019; Reitsema et al., 2018; Sand‐Jensen et al., 2008), invertebrates (Arzel et al., 2020; Vargas et al., 2022) and fish (Karlsson et al., 2009). Eutrophic boreal lakes, in particular, have become darker and more turbid during the last decades (de Wit et al., 2016; Holopainen & Lehikoinen, 2022), which may have negatively impacted populations at higher trophic levels (i.e. bottom‐up processes).

Invasive alien species affect native ecosystems through complex ecological interactions with native species (McGeoch et al., 2010), of which predation (through top‐down processes) is likely among those causing the most profound direct effects (Mooney & Cleland, 2001). Several invasive alien mammal predator species have become widely dispersed in Europe, such as the raccoon dog (*Nyctereutes procyonoides*) and the American mink (*Neovison vison*; hereafter mink) (Kauhala, 1996). Both species prefer habitats near wetlands and can have major adverse impacts on other vertebrates including amphibians, rodents, mustelids and ground‐nesting waterbirds (Bonesi & Palazon, 2007; Brzeziński et al., 2019; Koshev et al., 2020; Nummi et al., 2019). Waterbirds are ideal bioindicators for the ecological state of wetlands due to their dependence on the high‐quality of aquatic resources, as well as their role as prey for many native and alien predators. Long‐term waterbird surveys in Finland show alarming habitat‐specific population trajectories: populations on eutrophic lakes are declining more rapidly than those on oligotrophic lakes (Lehikoinen et al., 2016), possibly reflecting changes in water quality (Holopainen & Lehikoinen, 2022) or predation pressure (Pöysä et al., 2023; Pöysä & Linkola, 2021). The wetlands of northern Europe, including Finland, are highly important breeding areas for several waterbird species (Keller et al., 2020) and thus changes in these wetlands might have large‐scale effects on waterbird populations across Europe. Indeed, the ratio of juvenile ducks to adults in the harvest bag has declined in Britain, Denmark and Finland (Christensen & Fox, 2014; Mitchell et al., 2008; Pöysä & Väänänen, 2018), suggesting problems in reproductive success. It appears that harvest is not behind the declining trends, because both harvested and protected species are declining (Pöysä et al., 2013).

In the face of a multitude of stressors driving changes in wetland ecosystems, species‐specific outcomes, as deduced from changes in population trends, may vary depending on the species' ecological niche and/or on the trophic status of their preferred lakes. Currently, successful species are typically large and herbivorous, while declining species are smaller invertivorous species (Elmberg et al., 2020). Because successful management and conservation of wildlife populations requires detailed knowledge about limiting factors, we here set out to study whether changes in water chemistry (generating bottom‐up processes) and non‐native mammalian predator abundance can explain the diverging trends exhibited by various waterbirds in Finland. We hypothesise that while an increase in nutrient levels and darkening water colour affect all foraging niches negatively, invertivorous diving ducks and piscivores (i.e. visual predators) may have suffered the most from declines in foraging success. We also hypothesise that increases in pH benefit piscivorous ducks (Rask et al., 2014), but harm diving ducks: many fish species benefit from increasing pH, which means more food for piscivorous birds, but stronger competition between fish and invertivorous diving ducks (Nummi et al., 2016). Furthermore, we hypothesise that waterbird species nesting within or near wetlands are more vulnerable to predation by invasive alien predators than those nesting further away from the wetlands or in cavities (Holopainen et al., 2021; Pöysä et al., 2023; Pöysä & Linkola, 2021).

## 2 MATERIALS AND METHODS

### 2.1 Waterbird and habitat data

Voluntary‐based Finnish national waterbird surveys began in 1986, with the aim to monitor breeding bird abundance as measured in breeding pairs (Koskimies & Väisänen, 1991, Appendix S1). Two censuses of waterbird surveys were performed using standardised methods between late April and early June starting soon after the ice melts, which is gradually later towards higher latitudes (Koskimies & Väisänen, 1991). The pair numbers of the lakes are interpreted species specifically; early breeders during the first and late breeders during the latter survey. After a high effort in the first years (more than 1000 sites established), several sites were not covered annually. However, in 2020 and 2021, an effort was put into repeating the surveys at these sites. The data analysed here were collected during the years 1986–1989 and 2020–2021, hereafter referred to as periods 1 and 2, respectively. We here use the data from 17 common waterbird species, from 25,072 pairs (Table 1). To roughly evaluate whether climate change is systematically associated with the trends (e.g. climate change drives species distributions/phenology and causes trend‐associated shifts), we calculated the species‐specific mean weighted latitude of occurrence (MWLO: No. of Pairs*Wetland latitude/Tot. no. of Pairs) shifts between the periods (Brommer et al., 2012; Virkkala & Lehikoinen, 2014). We tested does the MWLO of species shifted significantly between period 1 and period 2 using a paired *t*‐test, where the null hypothesis was that the shift (i.e. difference between period 1 and period 2) was on average zero (function *t*‐test in R).

**TABLE 1:** Information for 17 waterbird species used in the analyses.

Species	Niche^a^	Nest site^b^	*NicheNest*	Mass (g)^c^	Hab. index^a^
Northern pintail *Anas acuta*	Dabbling invertivore	Near shore	2	700	1.68
Common teal *A. crecca*	Dabbling invertivore	Flexible	1	300	0.91
Mallard *A. platyrhynchos*	Dabbling invertivore	Flexible	1	975	1.28
Northern shoveler *Spatula clypeata*	Dabbling invertivore	Near shore	2	610	2.02
Eurasian wigeon *Mareca penelope*	Dabbling invertivore	Flexible	1	650	1.58
Common coot *Fulica atra*	Dabbl./diving invertivore	Wetland	3	900	2.89
Canada goose *Branta canadensis*	Herbivorous	Wetland	4	4350	1.43
Whooper swan *Cygnus cygnus*	Herbivorous	Wetland	4	9050	1.34
Common pochard *Aythya ferina*	Diving invertivore	Wetland	5	875	0.75
Tufted duck *A. fuligula*	Diving invertivore	Wetland	5	725	1.46
Common goldeneye *Bucephala clangula*	Diving invertivore	Cavity	6	800	0.62
Horned grebe *Podiceps auritus*	Diving invertivore	Wetland	5	560	1.24
Black‐throated loon *Gavia arctica*	Diving piscivore	Wetland	7	2350	−1.51
Common merganser *Mergus merganser*	Diving piscivore	Flexible	8	1600	−0.54
Red‐breasted merganser *M. serrator*	Diving piscivore	Flexible	8	1000	3.26
Great crested grebe *Podiceps cristatus*	Diving piscivore	Wetland	7	995	2.08
Red‐necked grebe *P. grisegena*	Diving piscivore	Wetland	7	845	2.51

*Note*: Niche is based on the foraging method and which type of food dominates the diet of a certain species during the breeding season. We used three classes: herbivore, invertivore, and piscivore (following classifications by Elmberg et al., 2020). Nest sites were determined based on the information given by Cramp et al. (1986): wetland nesters are nesting within wetlands or in immediate proximity of the wetlands and near‐shore nesters in the vicinity of wetlands, while flexible nesters can nest near or far away from wetlands (or in cavities) and cavity nesters nest solely in cavities. The common merganser (*M. merganser*) is classified as a flexible nester, while it also nests in cavities. Foraging niche and nest site preferences are combined (*NicheNest*) into eight categories (e.g. category 1 is flexible nesting dabbling ducks). *Mass* refers to the female weight (g). Habitat index describes the habitat preferences of the species from oligotrophic (negative end) to eutrophic (positive end) and is presented as background information. Differing from this table, the red‐breasted merganser (*Mergus serrator*) is in other sources described to indicate oligotrophic lakes (Kauppinen, 1993).

^a^ Elmberg et al. (2020).

^b^ Cramp et al. (1986).

^c^ Piha et al. (2019).

**FIGURE 1:**
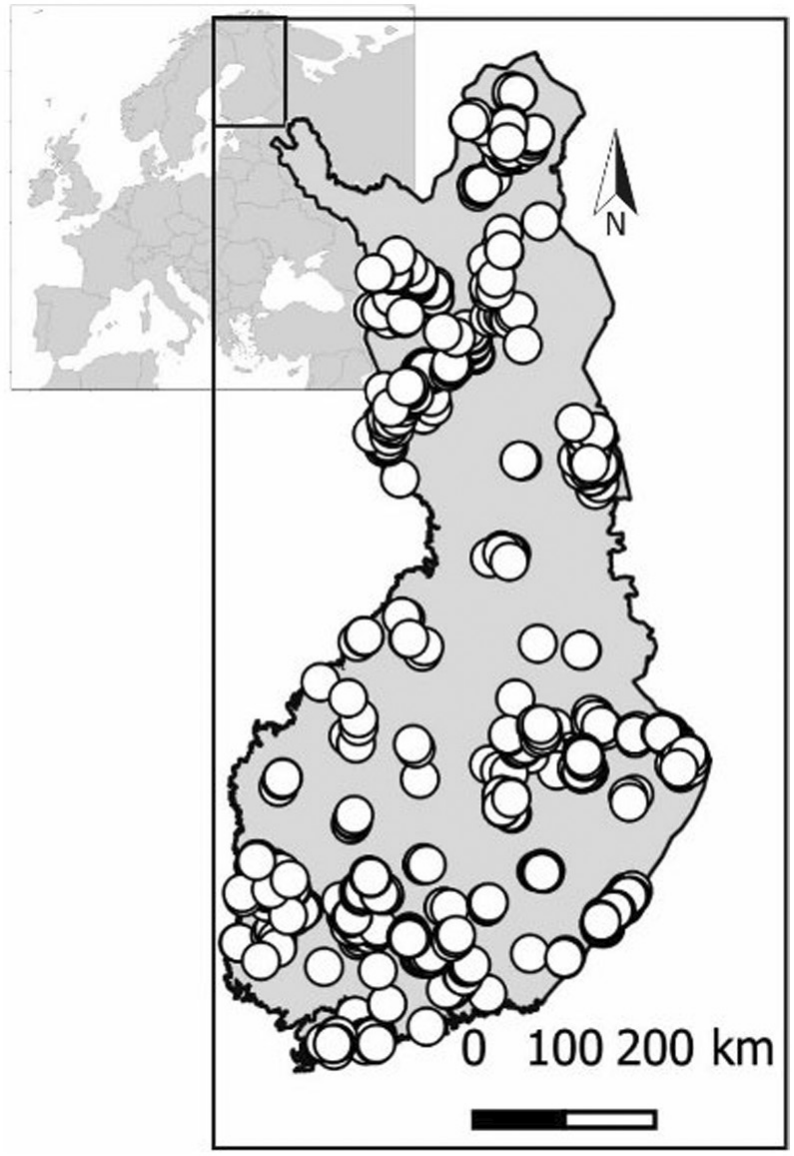
Location of Finland in Europe and the map of the 926 Finnish waterbird survey sites (habitat classes 1–4; Table S2) covered in both the 1980s and 2020s.

While surveying waterbirds, the volunteers also classified wetland habitat types based on vegetation, shoreline structure and water depth (Table S1). The main distinction is made between freshwater lakes and marine archipelago shores, in addition to the gradient from oligotrophic to eutrophic waters. Here we focus on lake habitats (classes 1–4 in Table S1; oligotrophic, semi‐mesotrophic, mesotrophic and eutrophic habitat type, respectively, see Holopainen & Lehikoinen, 2022). We only used sites that had been studied at least once during both periods, resulting in 926 sites that were distributed widely across Finland (Figure 1). In the analysis, we omitted sites for an individual species if it was never observed at the site to exclude sites that were not suitable for the focal species. In total, we had 907 sites with at least one waterbird observation (Table S2).

For the analyses, we transformed the four lake habitat classes into a two‐level factor. Based on earlier results (Holopainen & Lehikoinen, 2022; see also Figure S2), the reference factor class for nutrients, clarity and colour included the two most barren habitat classes (oligotrophic and semi‐mesotrophic; referred to as oligotrophic class in the analyses), which were then compared to the more nutrient‐rich classes (mesotrophic and eutrophic; referred as eutrophic class in the analyses). For the pH analysis, we combined the two most acidic classes (oligotrophic and mesotrophic) and compared them to the more alkaline classes (semi‐mesotrophic and eutrophic; Appendix S2).

To identify the possible reasons for the differing population trends between the waterbird species, we classified the species into groups based on foraging niche and nest site (Table 1). We assume that water chemistry can affect differently depending on the niche (e.g. water clarity affects foraging visibility of piscivore ducks; Holopainen et al., 2015) and nest site could expose the species to different levels of predation (e.g. species nesting at the shoreline are most threatened; Pöysä et al., 2023). We then combined niche and nest site types to form eight categories that represent different types of potential top‐down and bottom‐up responses (Table 1). When using categories in the generalised mixed models, one category will always be the reference to which the others are compared. Here we chose ‘flexible nesting dabbling ducks’ as the reference class in the main results because the population trends for these species are stable and they typically breed in all types of lakes. In an alternative model, we used ‘flexible nesting piscivores’ as a reference class (i.e. also stable population trends, breed mainly on oligotrophic lakes, results shown in Appendices S1–S4). Furthermore, we used female body mass as an explanatory variable to find out if smaller species are more defenceless against the alien predators than larger ones.

### 2.2 Physico‐chemical quality of the lakes

The systematic monitoring of lake water quality in Finland began in the 1960s (Kristensen & Bøgestrand, 1996). Finnish water chemistry measurements exhibit good spatial and temporal coverage. However, many of the waterbird monitoring lakes were not sampled at all or were not regularly sampled, resulting in temporally fragmented data. Based on earlier studies, we decided to focus on water chemistry parameters known to affect waterbirds (Holopainen et al., 2015). Therefore, we used total phosphorus and total nitrogen (μg/L, both unfiltered; hereafter phosphorus and nitrogen), pH, water colour (mgPt/L), and water clarity (Secchi depth) as explanatory variables in our analyses (hereafter: water chemistry variables). Data were extracted from the Open Data Service of the Finnish Environment Institute (Finnish Environment Institute VESLA, 2020; see details in Appendix S2). To combine the waterbird survey sites with appropriate water chemistry measurements, we created a 5‐km diameter (2.5 km radius) buffer around each survey site and included all the measurements performed within this buffer at the same lake. To increase the number of matching waterbird and water chemistry measurement surveys, we considered water chemistry measurements conducted during two 12‐year time windows (1986–1997 and 2010–2021). While the waterbird surveys and water chemistry data are not necessarily from the same years, we argue that these periods well enough reflect the overall water quality and its development over time for the purpose of this study. Using these 5‐km buffer zones and 12‐year time windows, we obtained measurements of water chemistry variables for waterbird survey sites as follows: phosphorus 141 (128 sites had waterbird observations: these sites were included in the analysis), nitrogen 127 (127), pH 142 (130), water clarity 130 (130) and water colour 125 (125). In total, we had 152 different waterbird survey sites with water chemistry measurements. We calculated site‐specific changes in water chemistry variables (averages for the measurement periods) from the first period to the second period. For every chemistry variable, we found both increasing and decreasing changes (Figure S3). As we are especially interested in the change and we can control the overall quality levels with habitat classes (i.e. oligotrophic and eutrophic or acidic and alkaline), in the analysis, period 1 was always given a value 0 and period 2 got the value of the change. Thus, change in water chemistry (Δ*Chemistry*) is a continuous variable that either increases or decreases from the base level set by the period 1. This allows us to study the association between changes in water chemistry and bird numbers at lakes differing in trophic status, even if the associations are opposing and trophic‐dependent.

Data exploration revealed that water chemistry measures were often strongly correlated (i.e. Pearson's correlation coefficient |*r*| > .6, Table S3). We omitted strongly correlated variables and used only total phosphorus, pH and colour in the models. To avoid making models too complicated, we analysed water chemistry variables in three different models.

### 2.3 Alien species data

The main invasive alien predators in Finland are the mink and the raccoon dog, both of which spread widely since the 1930s; currently, minks occupy the whole country, while raccoon dogs do not occur in the northernmost Lapland (Kauhala, 1996; Appendix S3). Especially, the raccoon dog seems to be a common duck nest predator on inland wetlands (Holopainen et al., 2021) and reaches especially high densities at eutrophic wetlands (Nummi et al., 2019). There are no population estimates for these species, but based on the harvest data, the mink population increased until the 1980s, where after the harvest bag size has stabilised. The raccoon dog harvest was at low levels until the 1980s, but has since increased dramatically (Figure S3). The national monitoring of the most important native nest predators (e.g. red fox *Vulpes vulpes*, hooded crow *Corvus corone* and Eurasian magpie *Pica pica*; Holopainen et al., 2020) suggests that the potential predation risk has more probably been declining than increasing during the last decades (Appendix S3).

Alien species data were based on the voluntarily reported harvest data on the ‘Oma riista’ – service from the years 2014 to 2021 (data from 282 wildlife management associations). We calculated annual average kills per hectare for each association for the period 2014–2021. We then created a 50 × 50 km grid to cover Finland, and for each grid cell, we calculated the mean annual harvest based on all associations overlapping with the grid. Applying this method we got a robust grid cell‐specific index for the invasive alien predator harvest. We assume that this index reflects the relative abundance of these predators within the grid cells (i.e. harvest rates reflect population density, see Ranta et al., 2008). We do not have similar indices available for the 1980s, but here we assume stable relative densities between the 1980s and the 2020s (i.e. areas have the same relative location along the index gradient in 1980s and 2020s). Therefore, we used the current index to measure geographical population densities in both study periods and by adding period interaction, we can find out whether the relative effect has changed in time (i.e. if the effect of predators has become stronger over time and ducks have declined more in the areas with high relative predator indices). We combined raccoon dog and mink indices for the analyses.

### 2.4 Statistics

The waterbird data were zero‐inflated, with overdispersion in the non‐zero part. We used zero‐inflated negative binomial models to accommodate both properties of the data (function glmmTMB in R, Brooks et al., 2017; R Development Core Team, 2021). Annual numbers of pairs of all the different waterbird species during periods 1 and 2 were used as the response variable (see Table 2 for all variables).

**TABLE 2:** Variables for the global predator and water chemistry models.

Model	Explanation
*Pairs*	Annual number of waterbird pairs observed at the survey site
*Lat*	Latitude, second‐order polynomial function, centralised
*Lon*	Longitude, centralised
*NicheNest*	Foraging type (four‐level factor: dabbling duck, herbivore, invertivore diving duck, piscivore) and Nest type (four‐level factor: flexible, shoreline, wetland) combined to a one eight‐level factor
*Pred. index*	Raccoon dog and American mink index for 50 × 50 km squares (mean annual harvest per hectare based on reports from wildlife management associations between 2014 and 2021)
*Mass*	Species‐specific waterbird female weight, centralised
*Hab*	Habitat class, a two‐level factor (trophic or acidity‐based)
*Period*	Period, continuous variable from −0.5 to 0.5 (period 1 = −0.5 and period 2 = 0.5)
Δ*Chemistry*	Water chemistry change (averaged over the years within a period) from the period 1 to 2 (for phosphorus, water colour, pH), centralised
*NicheNest: deltaChemistry*	Interaction: Water chemistry change (phosphorus, colour, pH) dependency on Niche(Nest) type
*Hab: deltaChemistry*	Interaction: Water chemistry change dependency on habitat class
*Predator index: Mass*	Interaction: Predator effect dependency on bird weight
*Predator index: Hab*	Interaction: Local‐scale predator occurrence
*Predator index: NicheNest*	Interaction: Predator effect dependency on (Niche)Nest type
*Period: Predator index*	Interaction: Predator effect in time
*Period: Weight*	Interaction: Weight effect in time
*Period: Hab*	Interaction: Habitat effect in time
*Period: NicheNest*	Interaction: NicheNest effect in time
*Period: Predator index: Weight*	Interaction: Weight effect in time in relation predator index
*Period: Predator index: Hab*	Interaction: Local‐scale predator occurrence effect in time
*Period:NicheNest*	Interaction: Nest(Niche) type vulnerability in relation to predator index in time
*Predator index*	
*Species*	Species as a factor (random part)
*Wetland ID*	Wetland ID as a factor (random part)

Species body mass (*Mass*, centralised), combined niche and nest types (*NicheNest*; an eight‐level factor) and lake habitat (*Hab*, a two‐level factor) were used as explanatory variables with lake latitude (*Lat*) and longitude (*Lon*; both centralised), alien predator index (*Pred Index*; centralised) and change in water chemistry (Δ*Chemistry*; centralised). As waterbird distribution may not change linearly along the long latitudinal gradient of Finland, we allowed latitude to take the shape of a second‐order polynomial function. Time period (*Period*) was converted to be a continuous dummy variable, where −0.5 represented period 1 and 0.5 represented period 2. Factor variables *Wetland ID* and *Species*, were included as random effects.

In addition to the main effects, we examined interactions that we identified a priori plausible. We tested interactions between (1) *Pred Index* and *Mass*, as larger waterbird species might defend their nests and broods more successfully and (2) *Pred Index* and *Hab*, as predator densities might be higher around eutrophic lakes (this effect was not included in the pH model due to a different habitat class approach), (3) *NicheNest* and Δ*Chemistry*, as water chemistry may have different effects on different waterbird niches, (4) *NicheNest* and *Pred index*, as nest site might affect nest depredation risk, and finally (5) *Hab* and Δ*Chemistry*, as chemical effects might be habitat class specific (i.e. an increase in nutrients in an oligotrophic lake would likely benefit production, while an increase in eutrophic lake might have negative effects). Furthermore, we examined two‐ and three‐way interactions with time (*Period*) to detect possible temporal changes. The interaction *Pred Index* × *NicheNest* × *Period* examined if the relative effect of alien predators changed through time (note that the variable *NicheNest* is a factor and thus the interpretation for this interaction is similar as for the two‐way interaction, but done *NicheNest*‐specifically; see Appendix S4 for the full model and graphical variable illustration).

We started each analysis with the full model and made model selection based on the *p*‐values: we first removed the higher order interactions until only significant *p*‐values remained or AIC‐values no longer improved, and then chose the most parsimonious model (Zuur et al., 2009). We, however, kept all those main effect terms, for which the variable of concern was included in significant interactions.

## 3 RESULTS

**FIGURE 2:**
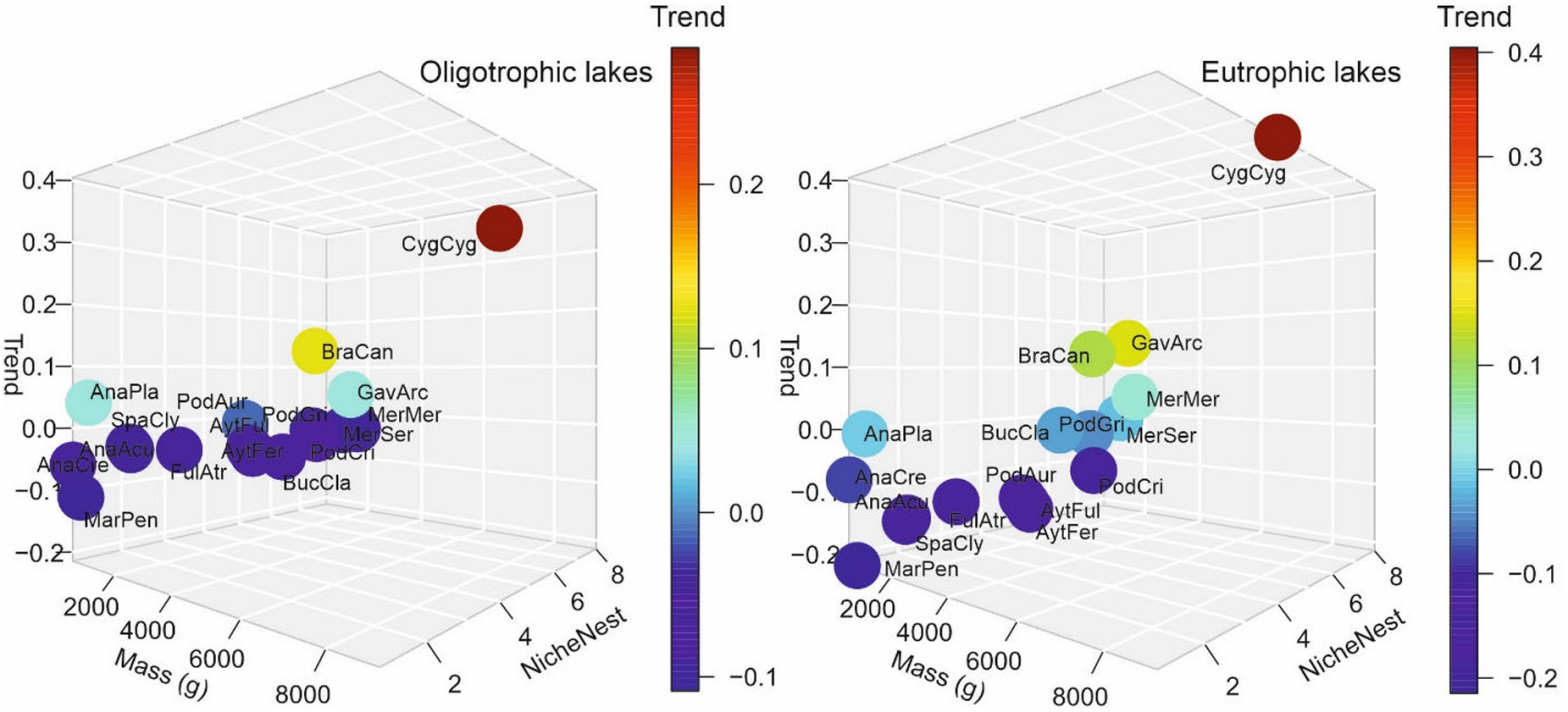
Specie‐specific trends from 1980s to 2020s (data from those lakes where species in question have been observed) in oligotrophic and eutrophic lakes in relation to species mass and niche. Species are indicated as abbreviations of their Latin names (see Table 1). *NicheNest*‐classification: dabbling ducks 1–3 (flexible nesting, nesting near‐shore nesting, nesting at wetlands; respectively); herbivores 4 (nesting at wetlands); diving ducks 5–6 (nesting at wetlands, nesting in cavities); piscivores 7–8 (nesting at wetlands, flexible nesting).

Our survey data from over 900 sites show that, in general, breeding numbers of Finnish waterbird species have declined since the 1980s. However, a common dabbling duck species, the mallard (*Anas platyrhynchos*), two large herbivorous species, that is, the whooper swan (*Cygnus cygnus*) and the non‐native Canada goose (*Branta canadensis*), and the piscivorous black‐throated loon (*Gavia arctica*), have increased and the common merganser's (*Mergus merganser*) population has remained stable (Figure 2, Table S4). Our findings, based on a limited number of years of bird census data, are in line with those from the continuous monitoring programme, indicating that our data well reflect the overall pattern of change in Finnish waterbird populations (Piha et al., 2022). Results across the species groups show that birds in all niches other than herbivores have declined (Table S4). Furthermore, populations of flexible nesters have remained stable, while species with other nesting types have decreased. Habitat‐specific results show that the declines occurred in all habitats but were milder at oligotrophic lakes. The mean weighted latitude of occurrence of species did not differ from zero (i.e. no significant shifts towards north or south) during the study period (*t*‐test, *t* = 0.68, df = 16, 95% CI = −24.60 to 47.90, *p* = .51). Furthermore, there was no correlation with the average MWLOs (average based on both periods) and species trends (Pearson correlation, *t* = −0.09, df = 15, 95% CI = −0.50 to 0.46, *p* = .93, *r* = −.02).

### 3.1 Water chemistry and predator modelling

Three primary models tested the associations between waterbird numbers and both the abundance of invasive alien predators and water chemistry (phosphorus, water colour and pH model; Tables S5–S7, see also results from alternative models in Tables S8–S10). Overall, the shared variables of the three models indicated that waterbird abundance was higher in the western parts of the country than in the east, and that bird abundance tended to be higher in the south than in the north. Phosphorus and water colour models suggested that there were more birds in the eutrophic habitats than in the oligotrophic ones (Tables S5 and S6), whereas the pH model showed that there were fewer birds in acidic lakes (Table S7) than in alkaline ones.

#### 3.1.1 Phosphorus

**FIGURE 3:**
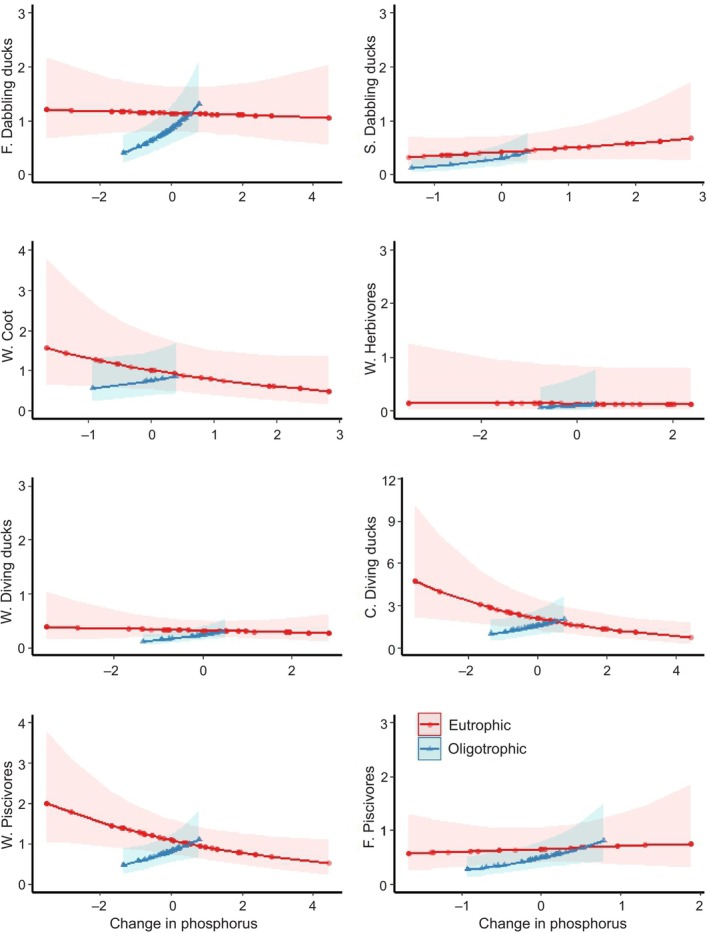
Predicted effects of changes to total phosphorus levels (μg/L, centralised) from period 1 (1980s, presented with the 0 level) to period 2 (2020s) on nest and niche‐specific waterbird pair abundances at eutrophic and oligotrophic lakes (fitted to generalized additive model). *y* axis: C, cavity nesting; F, flexible nesting; S, near‐shore nesting; W, wetland nesting. Hatched areas present 95% confidence bands.

Overall, increases in phosphorous were associated with higher waterbird abundances, but the effect seemed to be habitat‐dependent. Increased phosphorus levels were positively associated with bird numbers in oligotrophic lakes, whereas this effect was not detected in eutrophic lakes (Figure 3, Table S5). Compared to the reference group (flexible nesting dabbling ducks), increases in phosphorus were negatively associated with the abundance of the cavity nesting common goldeneye (*Bucephala clangula*) and potentially also on wetland nesting piscivores, however, the latter result was not statistically significant.

#### 3.1.2 Water colour

**FIGURE 4:**
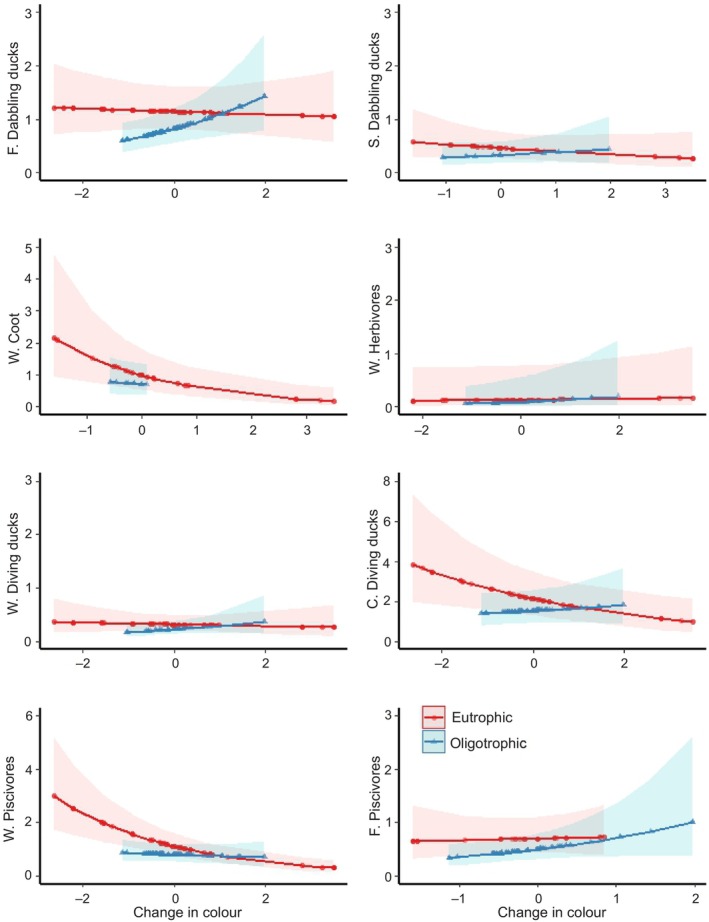
Predicted effects of changes to water colour levels (mgPt/L, centralised) from period 1 (1980s, presented with the 0 level) to period 2 (2020s) on nest and niche‐specific waterbird pair abundances at eutrophic and oligotrophic lakes (fitted to generalized additive model). *y* axis: C, cavity nesting; F, flexible nesting; S, near‐shore nesting; W, wetland nesting. Hatched areas present 95% confidence bands.

Similarly, as for phosphorus, browning of the water was positively associated with waterbird numbers, however, also this effect was habitat‐dependent and generally more positive in oligotrophic lakes than in eutrophic lakes (Figure 4, Table S6). At eutrophic lakes water browning was significantly negatively associated with common goldeneye, wetland nesting piscivore and common coot (*Fulica atra*) numbers, as compared to the reference group (flexible nesting dabbling ducks).

#### 3.1.3 pH

**FIGURE 5:**
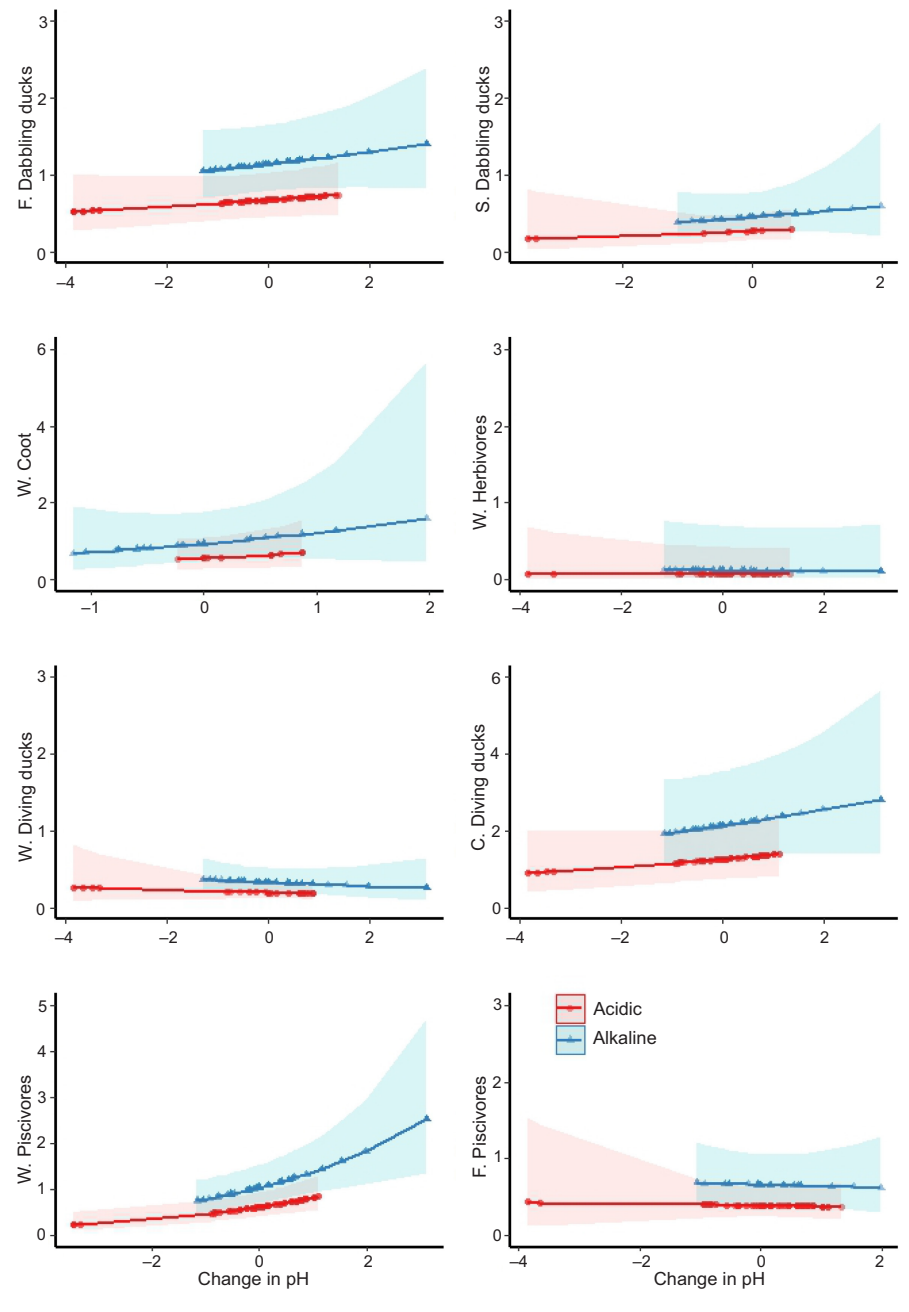
Predicted effects of changes to pH from period 1 (1980s, presented with the 0 level) to period 2 (2020s) on nest‐ and niche‐specific waterbird pair abundances at acidic and alkaline lakes (fitted to generalized additive model). *y* axis: C, cavity nesting; F, flexible nesting; S, near‐shore nesting; W, wetland nesting. Hatched areas present 95% confidence bands.

Waterbird abundance decreased over time in alkaline lakes as compared to acidic lakes. As hypothesised, the abundance of wetland nesting piscivores was associated positively with increased pH levels, as compared to the flexible nesting dabbling ducks (Figure 5, Table S7). The alternative model further suggested that wetland nesting piscivores may have been positively affected by increased pH levels, as compared to flexible nesting piscivores, but the result is only trend‐indicative (*p* = .086, Table S10).

#### 3.1.4 Invasive alien predators

Our results indicate that the association between the alien predator index and waterbird numbers was species‐specific (Tables S5–S7). The three‐way interaction with *Period*, *NicheNest* and *Predator index* revealed, as hypothesised, a significant negative association between invasive alien predators and wetland nesting diving ducks in time. These results indicate that the impact of predators (depicted by the predator index) on wetland nesting diving ducks has increased from period 1 to period 2. In addition, this three‐way interaction revealed a positive association between the predator index and wetland nesting herbivores and flexible nesting piscivores in time (the latter only in the pH model), as compared to flexible nesting dabbling ducks. These results indicate that wetland nesting herbivores and flexible nesting piscivores have been increasing especially in the areas with high predator indices. Furthermore, the alternative model, controlling the effects of pH, suggested a tendency that wetland nesting piscivores and the common goldeneye had declined more in concert with high predator abundance than flexible nesting piscivores, but these results are only trend‐indicative (*p* = .091 and *p* = .085, respectively, Table S10).

## 4 DISCUSSION

Invasive alien predators and changes in water quality appear to have asymmetric long‐term associations with waterbird populations over a large geographic area in Finland. The effects depend on the foraging niche of the waterbird species as well as on its nest site preferences. Additionally, the effects of changes in the water chemistry variables affected waterbirds differently depending on the trophic status of the wetlands. Furthermore, we did not detect a clear effect that the occurrence of species would have shifted northwards as would have been expected based on climate change scenarios (e.g. Virkkala & Lehikoinen, 2014), nor did we detect any associations between population trends and range shifts. We, therefore, argue that the wide decline of the boreal breeding waterbird species in Finland seems to be a consequence of both bottom‐up and top‐down processes at the wetlands driven by changes in water quality and the predator community, respectively, that are unrelated to climate change.

### 4.1 Bottom‐up associations

Eutrophic lakes have generally higher waterbird species richness and density than oligotrophic ones (Kauppinen, 1993; Nilsson, 1978; Paszkowski & Tonn, 2000). To some extent, nutrient enrichment may benefit waterbirds by increasing lake productivity, but negative effects might emerge in cases where excessive nutrient enrichment leads to eutrophication (Smith, 2003). Indeed, our study showed that an increase in phosphorus levels in oligotrophic lakes, but not in eutrophic ones, was positively related to waterbird numbers. A similar pattern was found regarding water colour, potentially reflecting the positive, but nonlinear relationship between dissolved organic carbon concentration (DOC, the most important factor affecting water colour in Finland; Arvola et al., 2010) and primary production in northern lakes. The positive relationship turns to a negative one when a certain DOC‐threshold level (4.8 mg/L) is exceeded, which is likely tied to light extinction (Seekell et al., 2015). We suggest that our habitat‐specific results emerge because this threshold level corresponds to a water colour level that lies between our oligotrophic and eutrophic habitat classes (Estlander et al., 2021; Holopainen & Lehikoinen, 2022).

Nutrient emissions from urban and farmland areas to lakes have led to the eutrophication of many freshwater ecosystems (Birk et al., 2020; Ekholm & Mitikka, 2006; Nilsson, 1978). Another apparent large‐scale source of emissions is peatland and forest ditching, which is an intensive forest management action that promotes erosion and sediment transport (Miettinen et al., 2020; Stenberg et al., 2015). It also shapes the hydrological connectivity between wetlands and their catchments (Covino, 2017). In Finland, forest ditching has been extensive: according to Holopainen and Lehikoinen (2022), the cumulative ditch length within a 5 km diameter buffer around waterbird survey sites varies between 0 and 373 km. The authors conclude that forest ditch abundance within catchments can be associated with the physico‐chemical status of the studied waterbird survey lakes (Holopainen & Lehikoinen, 2022).

We hypothesised that as visual hunters, diving ducks and piscivores would decline with lower visibility (Found et al., 2008; Kauppinen, 1993). Our results supported this hypothesis to some extent, but we suggest that the changes observed in water chemistry, especially concern species preferring eutrophic lakes (e.g. *Podiceps* spp.) and to a lesser extent species preferring oligotrophic lakes (e.g. mergansers; Kauppinen, 1993). Interestingly, also the common coot, a species using both diving and dabbling while foraging and which commonly prefers eutrophic habitats, seems to have suffered from browning water more than generalist dabbling ducks.

Diving ducks form an interesting group in our study and include the wetland‐nesting *Aythya* spp. and the cavity‐nesting common goldeneye. Earlier studies have attributed the decline of the two *Aythya* species in eutrophic lakes to resonate excessive eutrophication (e.g. Lehikoinen et al., 2016). However, of the diving duck species in this study, only the common goldeneye seemed to reflect the changes in phosphorus levels and water colour more than the reference group. It is possible that, although the common goldeneye has a diet similar to the *Aythya* spp., the effect of alien predators overrides the effects of water quality on wetland nesting *Aythya* species compared to the cavity nesting goldeneye. Similarly, Pöysä and Linkola (2021) suggested that the diverging population trends of *Aythya* spp. (declining in their study area) and the common goldeneye (increasing) are due to their differing nesting habits resulting in different effects of nest predation.

Studies focusing on the relationships between water quality and waterfowl abundance in boreal areas have produced equivocal results (Holopainen et al., 2015). Instead of direct effects, it is more probable that water characteristics influence the lower levels of the food web (i.e. generating varying bottom‐up effects). Thus, physico‐chemical changes might be mediated through complicated ecological interactions (Labaj et al., 2014; Langdon et al., 2006). Brownification has been found to have negative effects on aquatic invertebrate numbers and species composition at boreal lakes (Arzel et al., 2020). The causation behind the phenomenon is not clear, but light attenuation due to brownification has been observed to have a negative impact on water flea reproduction (Vargas et al., 2022). In boreal waters, brownification development might be detrimental for ducks, as food has been observed to limit the survival of mallard ducklings on boreal lakes (Gunnarsson et al., 2004). This notion is also supported by the fact that invertivore duck species, in particular, have shown large‐scale declines in Northern Europe (Elmberg et al., 2020).

As hypothesised, we found that an increase in pH was positively related to the abundances of wetland nesting piscivores (which mainly prefer eutrophic lakes), but not the flexibly nesting piscivores (i.e. mergansers, which prefer oligotrophic lakes). The pH levels of semi‐mesotrophic and eutrophic lakes (alkaline habitats in this study) have been increasing in Finland (Holopainen & Lehikoinen, 2022) as a result of a widespread recovery following emission reductions of sulphur since the 1980s (Stoddard et al., 1999). Increasing pH values especially affect fish that had declined when pH became too low, reducing their predation on invertebrates. While piscivorous waterbirds benefit from increased pH levels, diving invertivore ducks may suffer as a result of increased competition for food with fish (Nummi et al., 2012; Pöysä & Virtanen, 1994). Contrary to our hypothesis, we found no association between increased pH and invertivore duck numbers, but instead, our results show overall negative waterbird trends at alkaline lakes. In addition to increased fish‐duck competition, this might also reflect the interaction with sulphur deposition and the leaching of coloured organic substances leading to brownification (Arvola et al., 2010).

### 4.2 Top‐down associations

Several aspects of avian breeding biology, including nesting behaviour and annual offspring production are strongly shaped by predation (Holopainen et al., 2015). We found that the mode of nesting was indeed associated with the impact of invasive alien predators. Wetland nesting diving ducks seemed to have declined the most with the assumed increase of invasive alien predators. This finding likely reflects the fact that both the raccoon dog and the mink mainly hunt in the immediate proximity of the wetlands (Brzeziński et al., 2022, 2024; Holopainen et al., 2021; Kauhala, 1996). Since we used the same relative predator index for both time periods, our results indicate an increased effect by the invasive alien predators through time. Thus, nest predation caused by them is less likely to be compensatory and more likely to be additive (Holopainen et al., 2021).

Negative top‐down effects might also be exacerbated through species interactions. Earlier it has been shown that the loss of black‐headed gull (*Chroicocephalus ridibundus*) colonies reduces the protection of the other wetland nesting species, such as *Aythya* species and horned grebe (*Podiceps auritus*; Pöysä et al., 2019; Väänänen, 2000). According to Pöysä et al. (2019), it is possible that the presence of mink and raccoon dogs might be one cause of this decline in inland gull colonies.

The species classified as nearshore nesters show overall declines, not related to the predator indices, which might indicate that they suffer from some additional changes in the habitat that are not accounted for in our data. For instance, the reproductive success of northern pintails (*Anas acuta*) breeding on the North American prairies has drastically dropped due to changes in farming practices (Duncan & Devries, 2018). So far the other studies in Finland have found only mild, or lacking effects of land use changes on the breeding numbers of waterbirds (Hilli‐Lukkarinen et al., 2011).

While nesting at the wetlands, the largest species (i.e. swans, geese) are thriving, which may indicate that these species can potentially defend themselves against predators. Indeed, populations of both the whooper swan and the Canada goose have been increasing in southern Finland (Holopainen et al., 2022), where high predator index values occur. These herbivore species also forage on dry land and are possibly so able to avoid the negative effects of changes in water quality. Thus, their size and feeding habits may release these species from both negative top‐down and bottom‐up effects. Nevertheless, as shown by an earlier study, the whooper swan may in fact act as a habitat quality indicator and its increase has not had a negative effect on smaller waterbirds (Holopainen et al., 2022).

The only piscivore that has increased is the black‐throated loon, a heavy bird nesting in wetlands and exhibiting the lowest habitat index of all species. Also, flexible nesting piscivores (i.e. mergansers) showed to succeed better in high predator index areas than the reference group. We suggest that this is because oligotrophic habitats should experience the fewest negative changes considering both water chemistry (Holopainen & Lehikoinen, 2022) and invasive alien predators since high predator densities are observed especially in eutrophic environments (Nummi et al., 2019; Pöysä et al., 2023). This may contribute to the relative success of waterbirds inhabiting oligotrophic lakes (Holopainen et al., 2024). However, the predator index used in this study only describes the landscape‐level invasive alien predator abundance. More detailed studies are needed to show the differences in predator abundances between oligotrophic and eutrophic habitat types and their effects on waterbird populations.

We acknowledge that the diverging population trends of waterbirds might in some cases be driven by factors at work outside the breeding season. For instance, several factors affecting waterbirds during the wintering season (e.g. habitat loss and climate change) are suggested to be driving the general declining bird population trends in Europe (e.g. Burns et al., 2021; Pöysä & Väänänen, 2014). On the contrary, large herbivorous waterbirds are suggested to benefit from current farming practices in their wintering areas (Fox et al., 2017). However, we do not expect that factors working outside the breeding season could explain habitat‐specific population trajectories at the breeding sites.

### 4.3 Conservation implications

Long‐term breeding waterfowl surveys show drastic declining trends for several duck species in Finland over the last 30 years (Lehikoinen et al., 2016; Pöysä & Linkola, 2021). Our results support these findings emphasising waterbird losses, especially at eutrophic lakes, where drastic changes in water quality have taken place. In Europe, increased predation rates are possibly reducing the numbers of ground‐nesting birds, such as gamebirds and waders (MacDonald & Bolton, 2008; Roos et al., 2018). The increase in alien predator populations in Finland seems to have happened concurrently with the decline of many waterbird populations (Pöysä et al., 2023; Pöysä & Linkola, 2021). It may be necessary to control invasive alien predators around the lakes to enable breeding and brood production, especially of wetland nesting birds (Pöysä et al., 2023). Invasive alien predator removal has proven to be a cost‐effective and successful for seabird conservation measure (Jaatinen et al., 2022), and may indeed be a similarly effective and rapidly executed (while continuous) tool on wetlands. At the same time, water quality improvements are required. However, actions to improve water quality take time and are potentially demanding to execute. The effects of forest ditch coverage to the North European lakes are poorly studied, but as recently shown, forestry draining may contribute more to water quality than previously estimated (Nieminen, Palviainen, et al., 2018; Finér et al., 2021). It is crucial for managers to understand that the mitigation of negative effects on water quality requires restoration and management actions both at the wetland level (Lehikoinen et al., 2017) as well as at the level of the entire catchment area. Such measures need to focus on managing the hydrological connectivity between wetlands and their catchments (Heino et al., 2021; Shannon et al., 2019). Favouring forestry practices that lower harvest intensity and demand less ditch maintenance could potentially be an environmentally feasible management option (Nieminen, Hökkä, et al., 2018; Palviainen et al., 2022).

## AUTHOR CONTRIBUTIONS

**Sari Holopainen:** Conceptualization (lead); data curation (lead); formal analysis (lead); funding acquisition (equal); investigation (lead); methodology (lead); project administration (lead); resources (equal); software (equal); supervision (equal); validation (equal); visualization (lead); writing – original draft (lead); writing – review and editing (lead). **Kim Jaatinen:** Conceptualization (equal); data curation (equal); formal analysis (equal); funding acquisition (equal); investigation (equal); methodology (equal); project administration (supporting); resources (equal); software (equal); supervision (equal); validation (equal); visualization (supporting); writing – original draft (equal); writing – review and editing (equal). **Toni Laaksonen:** Conceptualization (equal); data curation (equal); formal analysis (equal); funding acquisition (equal); investigation (equal); methodology (equal); project administration (equal); resources (equal); software (equal); supervision (equal); validation (equal); visualization (equal); writing – original draft (equal); writing – review and editing (equal). **Andreas Lindén:** Conceptualization (equal); data curation (equal); formal analysis (lead); funding acquisition (equal); investigation (equal); methodology (lead); project administration (equal); resources (equal); software (lead); supervision (equal); validation (equal); visualization (lead); writing – original draft (equal); writing – review and editing (equal). **Petri Nummi:** Conceptualization (supporting); data curation (supporting); formal analysis (supporting); funding acquisition (supporting); investigation (supporting); methodology (supporting); project administration (supporting); resources (supporting); software (supporting); supervision (supporting); validation (supporting); visualization (supporting); writing – original draft (supporting); writing – review and editing (supporting). **Markus Piha:** Conceptualization (supporting); data curation (supporting); formal analysis (supporting); funding acquisition (supporting); investigation (supporting); methodology (supporting); project administration (supporting); resources (supporting); software (supporting); supervision (supporting); validation (supporting); visualization (supporting); writing – original draft (supporting); writing – review and editing (supporting). **Hannu Pöysä:** Conceptualization (equal); data curation (equal); formal analysis (equal); funding acquisition (equal); investigation (equal); methodology (equal); project administration (equal); resources (equal); software (equal); supervision (equal); validation (equal); visualization (equal); writing – original draft (equal); writing – review and editing (equal). **Tero Toivanen:** Conceptualization (supporting); data curation (supporting); formal analysis (supporting); funding acquisition (equal); investigation (supporting); methodology (supporting); project administration (supporting); resources (supporting); software (supporting); supervision (supporting); validation (supporting); visualization (supporting); writing – original draft (supporting); writing – review and editing (supporting). **Veli‐Matti Väänänen:** Conceptualization (supporting); data curation (supporting); formal analysis (supporting); funding acquisition (supporting); investigation (supporting); methodology (supporting); project administration (supporting); resources (supporting); software (supporting); supervision (supporting); validation (supporting); visualization (supporting); writing – original draft (supporting); writing – review and editing (supporting). **Mikko Alhainen:** Conceptualization (supporting); data curation (equal); formal analysis (supporting); funding acquisition (supporting); investigation (supporting); methodology (supporting); project administration (supporting); resources (supporting); software (supporting); supervision (supporting); validation (supporting); visualization (supporting); writing – original draft (supporting); writing – review and editing (supporting). **Aleksi Lehikoinen:** Conceptualization (lead); data curation (lead); formal analysis (equal); funding acquisition (lead); investigation (lead); methodology (lead); project administration (lead); resources (lead); software (lead); supervision (lead); validation (lead); visualization (equal); writing – original draft (equal); writing – review and editing (equal).

## FUNDING INFORMATION

This study was funded by The Finnish Ministry of Agriculture and Forestry (grant 1440/03.02.04.00/2019), Metsähallitus and Nature and Game Management Trust Finland. AL received funding from the Research Council of Finland (grants 323527 and 329251).

## CONFLICT OF INTEREST STATEMENT

We declare that the authors of this article have no conflicts of interest.

## Supporting information


Appendix S1.

## Data Availability

The waterfowl survey data used in this study are available in the open‐access data repository maintained by the Finnish Biodiversity Information Facility (FinBIF). Water chemistry data is freely available on the Finnish Environmental Centre Open data Web service (Hertta). Alien predator data is collected by the Finnish Wildlife Agency. The agency is responsible for data deposition.
