# Supplementary material for: Anthropogenic bottom‐up and top‐down impacts on boreal breeding waterbirds
Source: Ecol Evol. 2024 Mar 10;14(3):e11136. doi: 10.1002/ece3.11136 (PMC10925514; doi:10.1002/ece3.11136)
Supplement: Supplementary file 1 — Appendix S1. [file ECE3-14-e11136-s001.docx]

**Anthropogenic bottom-up and top-down impacts on boreal breeding waterbirds**

Sari Holopainen^1,2I^, Kim Jaatinen^3^, Toni Laaksonen^4^, Andreas Lindén^5^, Petri Nummi^2^, Markus Piha^5^, Hannu Pöysä^6,7^, Tero Toivanen^8^, Veli-Matti Väänänen^2^, Mikko Alhainen^9^ & Aleksi Lehikoinen^1^

1. Luonnontieteellinen keskusmuseo, Finnish Museum of Natural History, P.O. Box 17, 00014 University of Helsinki, Finland. Corresponding author: Sari Holopainen sari.holopainen@helsinki.fi. Orcid: 0000-0002-3271-4468. aleksi.lehikoinen@helsinki.fi
2. Department of Forest Sciences, P.O. Box 27, FI-00014 University of Helsinki, Finland. petri.nummi@helsinki.fi, veli-matti.vaananen@helsinki.fi
3. Nature and Game Management Trust Finland, Degerbyvägen 176, FI-10160, Degerby, Finland. kim@luontojariista.fi
4. Department of Biology, University of Turku, FI-20014 Turku, Finland. tokrla@utu.fi
5. Natural Resources Institute Finland Luke, Latokartanonkaari 9, 00790 Helsinki, Finland. andreas.linden@luke.fi, markus.piha@luke.fi
6. Natural Resources Institute Finland Luke, Yliopistokatu 6, 80100 Joensuu, Finland. hannu.poysa@outlook.com
7. University of Eastern Finland, Department of Environmental and Biological Sciences, POB 111, FI-80101 Joensuu, Finland. hannu.poysa@outlook.com
8. BirdLife Finland, Annankatu 29 A 16, 00100 Helsinki, Finland. tero.toivanen@birdlife.fi
9. Finnish Wildlife Agency, Sompiontie 1, 00730 Helsinki, Finland. mikko.alhainen@riista.fi
10. Present address

**Supplementary material**

Table A1. Habitat classes used to define wetlands in the Finnish waterbird surveys 1986 onwards. Classes 9 and 10 were added in (2020). The class names used in this study are indicated in italics (see Holopainen and Lehikoinen, 2021).

| Class | Description |
| --- | --- |
|  |  |
| 1 | Oligotrophic lake surrounded by forest or peatland (black-throated diver *Gavia arctica* and mergansers Mergus sp. are indicator species in lakes over 1 km^2^). *Oligotrophic lakes* |
| 2 | Deep lake with reed *Phragmites australis* beds in bays (great crested grebe *Podiceps cristatus* as an indicator in southern Finland). *Semi-mesotrophic lakes* |
| 3 | Shallow lake with forest or peatland shore with luxuriant emergent vegetation (brown water, indicator Equisetum, high bird density, water level can be lowered): *Mesotrophic* |
| 4 | Lake with luxuriant vegetation within agricultural landscape or near human settlements (rather shallow, typically good bird lake with abundant reed beds; more common in southern and southwest Finland). *Eutrophic lakes* |
| 5 | Oligotrophic seashore with barren vegetation. *Oligotrophic seashore* |
| 6 | Eutrophic seashore with some reed beds (both deep and shallow shores). *Semi-eutrophic seashore* |
| 7 | Seashore with luxuriant vegetation (both deep and shallow shores). *Eutrophic seashore* |
| 8 | Other, e.g. river and peatland. *Others* |
| 9 | Overgrown or destroyed. *Unsuitable* |
| 10 | Constructed wetland. *Constructed* |

Table A2. The species-specific number of sites and pair observations in survey sites with phosphorus, water color and pH measurements. “N. of sites” indicates the number of survey sites the species was observed at least once during the study years 1986-1989 and 2020-202. “*N. of P/Col./pH sites*” indicates the number of survey sites with water chemistry measurements. “*N. of pairs P/Col./pH*” sums up the number of pairs observed at the sites. P = total phosphorus, col = colour.

| Species | N. of sites | N. of P sites | N. of col. sites | N. of pH sites | N. of pairs P | N. of pairs col. | N. of pairs pH |
| --- | --- | --- | --- | --- | --- | --- | --- |
| *Anas acuta* | 132 | 24 | 22 | 23 | 94 | 87 | 88 |
| *A. crecca* | 600 | 98 | 94 | 97 | 705 | 654 | 679 |
| *A. platyrhynchos* | 646 | 109 | 105 | 109 | 956 | 904 | 934 |
| *Spatula clypeata* | 90 | 19 | 18 | 18 | 148 | 129 | 129 |
| *Mareca penelope* | 432 | 79 | 77 | 78 | 482 | 461 | 472 |
| *Fulica atra* | 98 | 35 | 33 | 34 | 411 | 385 | 386 |
| *Branta canadensis* | 48 | 17 | 17 | 17 | 22 | 22 | 22 |
| *Cygnus gygnus* | 353 | 66 | 63 | 67 | 119 | 114 | 120 |
| *Aythya ferina* | 110 | 30 | 29 | 29 | 183 | 172 | 172 |
| *A. fuligula* | 293 | 51 | 49 | 50 | 329 | 312 | 315 |
| *Bucephala clangula* | 755 | 118 | 114 | 118 | 1338 | 1293 | 1350 |
| *Podiceps auritus* | 57 | 11 | 11 | 11 | 56 | 56 | 56 |
| *Gavia arctica* | 339 | 62 | 59 | 62 | 192 | 186 | 192 |
| *Mergus merganser* | 240 | 63 | 60 | 63 | 190 | 185 | 194 |
| *M. serrator* | 171 | 48 | 47 | 49 | 166 | 165 | 169 |
| *Podiceps cristatus* | 230 | 79 | 76 | 78 | 1062 | 986 | 1046 |
| *P. grisegena* | 106 | 39 | 38 | 39 | 143 | 142 | 143 |

Table A3. Spearman correlation for water chemistry measurement at waterbird survey sites (phosphorus 141 sites, nitrogen 127 sites, pH 142 sites, water clarity 130 sites and water colour 125 sites; results averaged per site per period) for the two study periods (1986-1997 and 2010-2021).

|  | Total phosphorus | Total nitrogen | pH | Clarity |
| --- | --- | --- | --- | --- |
| *Period 1* |  |  |  |  |
| Total phosphorus | - |  |  |  |
| Total nitrogen | 0.9 | - |  |  |
| pH | 0.3 | 0.2 | - |  |
| Clarity | -0.8 | -0.7 | -0.2 | - |
| Colour | 0.5 | 0.5 | -0.3 | -0.5 |
| *Period 2* |  |  |  |  |
| Total phosphorus | - |  |  |  |
| Total nitrogen | 0.9 | - |  |  |
| pH | 0.3 | 0.2 | - |  |
| Clarity | -0.7 | -0.7 | 0.0 | - |
| Colour | 0.5 | 0.5 | -0.4 | -0.5 |

Table A4. Pearson correlations (*r*) for pairs and year from 1980s to 2020s (data from all the waterbird survey lakes with species and qualities in question: lakes omitted species-specifically if not observed at that site) presented for i) species-specifically, ii) *NicheNest*-specifically and iii) habitat-specifically. For habitats the results are shown at lake-level (i.e. waterbirds summed together on lake-level), but others are analysed on species and lake-level. See Table 1 for *NiceNest*-classifications and Table A1 for the habitat classifications. *N* indicates the total number of pairs observed during the study periods within the group in question. Significant test values (*P* < 0.05) are in bold. MWLO shift measures a species-specific mean weighted latitude of occurrence shift in kilometres from 1980s to 2020s: minus = southward, plus = northwards.

| Species | *N* | *t* | df | *r* | *P* | MWLO  shift (km) |
| --- | --- | --- | --- | --- | --- | --- |
| ***Anas acuta*** | **458** | **-4.71** | **582** | **-0.19** | **< 0.001** | -50 |
| ***A. crecca*** | **3 236** | **-2.60** | **2 499** | **-0.05** | **0.009** | -3 |
| ***A. platyrhynchos*** | **3 732** | **2.26** | **2 720** | **0.04** | **0.024** | 19 |
| ***Spatula clypeata*** | **515** | **-4.29** | **4 11** | **-0.21** | **< 0.001** | 25 |
| ***Mareca penelope*** | **1 905** | **-8.46** | **1 804** | **-0.20** | **< 0.001** | 18 |
| ***Fulica atra*** | **1 249** | **-4.74** | **430** | **-0.22** | **< 0.001** | -14 |
| ***Branta canadensis*** | **81** | **8.76** | **205** | **0.52** | **< 0.001** | 88 |
| ***Cygnus cygnus*** | **607** | **26.41** | **1 471** | **0.57** | **< 0.001** | -140 |
| ***Aythya ferina*** | **686** | **-6.07** | **498** | **-0.26** | **< 0.001** | -32 |
| ***A. fuligula*** | **1 903** | **-5.79** | **1 226** | **-0.16** | **< 0.001** | 157 |
| ***Bucephala clangula*** | **5 232** | **-2.98** | **3 145** | **-0.05** | **0.003** | 35 |
| ***Podiceps auritus*** | **313** | **-4.68** | **261** | **-0.28** | **< 0.001** | 99 |
| ***Gavia arctica*** | **730** | **3.59** | **1 439** | **0.09** | **< 0.001** | -69 |
| *Mergus merganser* | 641 | -0.44 | 1 022 | -0.01 | 0.659 | -53 |
| ***M. serrator*** | **423** | **-2.22** | **700** | **-0.08** | **0.027** | 69 |
| ***Podiceps cristatus*** | **2 945** | **-3.81** | **996** | **-0.12** | **< 0.001** | 13 |
| ***P. grisegena*** | **416** | **-2.29** | **446** | **-0.11** | **0.022** | 36 |
| Nest (flexible) | 8 032 | -0.55 | 6 947 | -0.00 | 0.582 |  |
| **Nest (shore)** | **2 878** | **-10.55** | **2 801** | **-0.20** | **< 0.001** |  |
| **Nest (wetland)** | **8 930** | **-6.68** | **6 988** | **-0.08** | **< 0.001** |  |
| **Nest (cavity)** | **5 232** | **-2.98** | **3 145** | **-0.05** | **0.003** |  |
| **Niche (dabbling ducks)** | **11 095** | **-6.86** | **8 456** | **-0.07** | **< 0.001** |  |
| **Niche (herbivores)** | **688** | **27.65** | **1 678** | **0.56** | **< 0.001** |  |
| **Niche (diving ducks)** | **8 134** | **-8.40** | **5 136** | **-0.12** | **< 0.001** |  |
| **Niche (piscivores)** | **5 155** | **-3.91** | **4 611** | **-0.06** | **< 0.001** |  |
| *Habitat (Oligo-Lake)* | *6 410* | *-1.93* | *2 186* | *-0.04* | *0.053* |  |
| **Habitat (S-Meso-Lake)** | **4 962** | **-2.40** | **475** | **-0.11** | **< 0.017** |  |
| **Habitat (Meso-Lake)** | **4 089** | **-5.21** | **503** | **-0.23** | **< 0.001** |  |
| **Habitat (Eu-Lake)** | **9 611** | **-3.00** | **561** | **-0.13** | **< 0.001** |  |

Table A5. Parameters for the alien predator and phosphorus model. Intercept is representing *NicheNest* (flexible nesting dabbling ducks) and *Hab* (Oligotrophic). *Pred. index* = predator index, *ΔP* = the change in the phosphorus levels between the study periods. See the variable explanations in the table 2. Dispersion parameter for nbinom2 family = 1.44. Random effect variances: *sp* = 0.07, *SiteID* = 0.38. Significant test values (P < 0.05) are bolded, and trend-setting values (P < 0.1) are shown in italics.

|  | Estimate | SE | z-value | P |
| --- | --- | --- | --- | --- |
| Intercept | -0.167 | 0.206 | -0.810 | 0.418 |
| poly(Lat)1 | 11.114 | 8.080 | 1.375 | 0.169 |
| *poly(Lat)2* | *-10.139* | *5.248* | *-1.932* | *0.053* |
| **Lon** | **-0.126** | **0.050** | **-2.527** | **0.011** |
| Pred. index | 0.125 | 0.148 | 0.841 | 0.400 |
| Mass | -0.364 | 0.233 | -1.563 | 0.118 |
| **Hab (eutrophic)** | **0.297** | **0.102** | **2.902** | **0.004** |
| **ΔP** | **0.544** | **0.170** | **3.200** | **0.001** |
| **NicheNest (dabbl. near shore)** | **-1.007** | **0.283** | **-3.554** | **<0.001** |
| NicheNest (dabbl. wetland) | -0.115 | 0.335 | -0.342 | 0.732 |
| **NicheNest (herbiv. wetland)** | **-2.124** | **0.977** | **-2.173** | **0.030** |
| **NicheNest (diving wetland)** | **-1.278** | **0.259** | **-4.937** | **<0.001** |
| *NicheNest (diving cavity)* | *0.607* | *0.320* | *1.898* | *0.058* |
| NicheNest (pisciv. wetland) | -0.037 | 0.250 | -0.147 | 0.883 |
| **NicheNest (pisciv. flexible)** | **-0.553** | **0.268** | **-2.068** | **0.039** |
| **Period** | **0.439** | **0.118** | **3.711** | **<0.001** |
| Pred. index: NicheNest (dabbl. near shore) | -0.235 | 0.163 | -1.442 | 0.149 |
| *Pred. index: NicheNest (dabbl. wetland)* | *0.248* | *0.144* | *1.725* | *0.084* |
| **Pred. index: NicheNest (herbiv. wetland)** | **-1.254** | **0.547** | **-2.291** | **0.022** |
| **Pred. index: NicheNest (diving wetland)** | **-0.489** | **0.143** | **-3.421** | **0.001** |
| Pred. index: NicheNest (diving cavity) | -0.075 | 0.069 | -1.091 | 0.275 |
| Pred. index: NicheNest (pisciv. wetland) | 0.088 | 0.069 | 1.267 | 0.205 |
| **Pred. index: NicheNest (pisciv. flexible)** | **-0.218** | **0.091** | **-2.387** | **0.017** |
| **Hab (eutrophic): ΔP** | **-0.561** | **0.170** | **-3.300** | **0.001** |
| NicheNest (dabbl. near shore): ΔP | 0.188 | 0.167 | 1.122 | 0.262 |
| NicheNest (dabbl. wetland): ΔP | -0.239 | 0.173 | -1.377 | 0.169 |
| NicheNest (herbiv. wetland): ΔP | -0.022 | 0.135 | -0.159 | 0.873 |
| NicheNest (diving wetland): ΔP | -0.039 | 0.154 | -0.256 | 0.798 |
| **NicheNest (diving cavity): ΔP** | **-0.215** | **0.090** | **-2.401** | **0.016** |
| *NicheNest (pisciv. wetland): ΔP* | *-0.154* | *0.088* | *-1.754* | *0.079* |
| NicheNest (pisciv. flexible): ΔP | 0.095 | 0.211 | 0.452 | 0.651 |
| Period: Pred. index | 0.007 | 0.084 | 0.088 | 0.930 |
| **Period: Mass** | **1.106** | **0.217** | **5.105** | **<0.001** |
| Period: NicheNest (dabbl. near shore) | -0.389 | 0.267 | -1.458 | 0.145 |
| **Period: NicheNest (dabbl. wetland)** | **-0.718** | **0.236** | **-3.046** | **0.002** |
| Period: NicheNest (herbiv. wetland) | 2.042 | 1.398 | 1.461 | 0.144 |
| **Period: NicheNest (diving wetland)** | **-2.036** | **0.244** | **-8.342** | **<0.001** |
| Period: NicheNest (diving cavity) | -0.031 | 0.132 | -0.236 | 0.813 |
| **Period: NicheNest (pisciv. wetland)** | **-0.591** | **0.141** | **-4.207** | **<0.001** |
| Period: NicheNest (pisciv. flexible) | -0.040 | 0.177 | -0.224 | 0.823 |
| Period: NicheNest (dabbl. near shore): Pred. index | -0.020 | 0.310 | -0.064 | 0.949 |
| Period: NicheNest (dabbl. wetland): Pred. index | 0.217 | 0.281 | 0.771 | 0.441 |
| **Period: NicheNest (herbiv. wetland): Pred. index** | **2.356** | **1.088** | **2.166** | **0.030** |
| **Period: NicheNest (diving wetland): Pred. index** | **-1.081** | **0.278** | **-3.880** | **<0.001** |
| Period: NicheNest (diving cavity): Pred. index | 0.032 | 0.136 | 0.234 | 0.815 |
| Period: NicheNest (pisciv. wetland): Pred. index | 0.093 | 0.136 | 0.683 | 0.494 |
| Period: NicheNest (pisciv. flexible): Pred. index | 0.263 | 0.175 | 1.501 | 0.133 |
| Zero-inflation model |  |  |  |  |
| **Intercept** | **-3.119** | **0.636** | **-4.903** | **<0.001** |

Table A6. Parameters for the alien predator and water color model. Intercept is representing *NicheNest* (flexible nesting dabbling ducks) and *Hab* (Oligotrophic). *Pred. index* = predator index, *ΔCol* = the change in the color value between the study periods. See the variable explanations in the table 2. Dispersion parameter for nbinom2 family = 1.41. Random effect variances: *sp* = 0.08; *SiteID* = 0.40. Significant test values (P < 0.05) are bolded, and trend-setting values (P < 0.1) are shown in italics.

|  | Estimate | SE | z-value | P |
| --- | --- | --- | --- | --- |
| Intercept | -0.203 | 0.198 | -1.023 | 0.306 |
| poly(Lat)1 | 7.445 | 8.100 | 0.919 | 0.358 |
| poly(Lat)2 | -7.824 | 5.148 | -1.520 | 0.129 |
| **Lon** | **-0.123** | **0.051** | **-2.422** | **0.015** |
| Pred. index | 0.105 | 0.152 | 0.690 | 0.490 |
| Mass | -0.346 | 0.225 | -1.534 | 0.125 |
| **Hab (eutrophic)** | **0.335** | **0.107** | **3.121** | **0.002** |
| **ΔCol** | **0.284** | **0.111** | **2.552** | **0.011** |
| **NicheNest (dabbl. near shore)** | **-0.916** | **0.268** | **-3.414** | **0.001** |
| NicheNest (dabbl. wetland) | -0.135 | 0.319 | -0.424 | 0.671 |
| **NicheNest (herbiv. wetland)** | **-2.167** | **0.958** | **-2.263** | **0.024** |
| **NicheNest (diving wetland)** | **-1.274** | **0.249** | **-5.126** | **<0.001** |
| **NicheNest (diving cavity)** | **0.627** | **0.301** | **2.083** | **0.037** |
| NicheNest (pisciv. wetland) | -0.029 | 0.237 | -0.124 | 0.901 |
| *NicheNest (pisciv. flexible)* | *-0.493* | *0.255* | *-1.938* | *0.053* |
| **Period** | **0.386** | **0.122** | **3.161** | **0.002** |
| Pred. index: NicheNest (dabbl. near shore) | -0.149 | 0.161 | -0.928 | 0.353 |
| Pred. index: NicheNest (dabbl. wetland) | 0.246 | 0.160 | 1.542 | 0.123 |
| **Pred. index: NicheNest (herbiv. wetland)** | **-1.275** | **0.529** | **-2.410** | **0.016** |
| **Pred. index: NicheNest (diving wetland)** | **-0.529** | **0.140** | **-3.790** | **<0.001** |
| Pred. index: NicheNest (diving cavity) | -0.108 | 0.071 | -1.520 | 0.128 |
| Pred. index: NicheNest (pisciv. wetland) | 0.093 | 0.073 | 1.285 | 0.199 |
| **Pred. index: NicheNest (pisciv. flexible)** | **-0.259** | **0.093** | **-2.790** | **0.005** |
| **Hab (eutrophic): ΔCol** | **-0.307** | **0.117** | **-2.632** | **0.008** |
| NicheNest (dabbl. near shore): ΔCol | -0.128 | 0.154 | -0.831 | 0.406 |
| **NicheNest (dabbl. wetland): ΔCol** | **-0.457** | **0.167** | **-2.738** | **0.006** |
| NicheNest (herbiv. wetland): ΔCol | 0.089 | 0.135 | 0.657 | 0.511 |
| NicheNest (diving wetland): ΔCol | -0.030 | 0.151 | -0.196 | 0.845 |
| **NicheNest (diving cavity): ΔCol** | **-0.198** | **0.093** | **-2.121** | **0.034** |
| **NicheNest (pisciv. wetland): ΔCol** | **-0.354** | **0.094** | **-3.764** | **<0.001** |
| NicheNest (pisciv. flexible): ΔCol | 0.068 | 0.203 | 0.337 | 0.736 |
| Period: Pred. index | 0.055 | 0.088 | 0.625 | 0.532 |
| **Period: Mass** | **1.050** | **0.224** | **4.687** | **<0.001** |
| Period: NicheNest (dabbl. near shore) | -0.225 | 0.260 | -0.865 | 0.387 |
| **Period: NicheNest (dabbl. wetland)** | **-0.754** | **0.249** | **-3.026** | **0.002** |
| Period: NicheNest (herbiv. wetland) | 2.257 | 1.413 | 1.597 | 0.110 |
| **Period: NicheNest (diving wetland)** | **-2.072** | **0.252** | **-8.225** | **<0.001** |
| *Period: NicheNest (diving cavity)* | -0.045 | 0.136 | -0.331 | 0.741 |
| **Period: NicheNest (pisciv. wetland)** | **-0.597** | **0.147** | **-4.065** | **<0.001** |
| Period: NicheNest (pisciv. flexible) | 0.012 | 0.188 | 0.065 | 0.949 |
| Period: NicheNest (dabbl. near shore): Pred. index | 0.074 | 0.301 | 0.245 | 0.807 |
| Period: NicheNest (dabbl. wetland): Pred. index | 0.190 | 0.312 | 0.609 | 0.543 |
| **Period: NicheNest (herbiv. wetland): Pred. index** | **2.239** | **1.051** | **2.131** | **0.033** |
| **Period: NicheNest (diving wetland): Pred. index** | **-1.145** | **0.271** | **-4.224** | **<0.001** |
| Period: NicheNest (diving cavity): Pred. index | 0.008 | 0.141 | 0.058 | 0.954 |
| Period: NicheNest (pisciv. wetland): Pred. index | 0.089 | 0.142 | 0.622 | 0.534 |
| *Period: NicheNest (pisciv. flexible): Pred. index* | *0.305* | *0.178* | *1.712* | *0.087* |
| Zero-inflation model |  |  |  |  |
| **Intercept** | **-3.153** | **0.729** | **-4.326** | **<0.001** |

Table A7. Parameters for the alien predator and pH model. Intercept is representing *NicheNest* (flexible nesting dabbling ducks) and *Hab* (Acid.). *Pred. in*dex = predator index, *ΔpH* = the change in the pH between the study periods. See the variable explanations in the table 2. Dispersion parameter for nbinom2 family = 1.41. Random effect variances: *sp* = 0.07, *SiteID* = 0.41. Significant test values (P < 0.05) are bolded, and trend-setting values (P < 0.1) are shown in italics.

|  | Estimate | SE | z-value | P |
| --- | --- | --- | --- | --- |
| *Intercept* | *-0.397* | *0.213* | *-1.870* | *0.061* |
| *poly(Lat)1* | *16.214* | *8.277* | *1.959* | *0.050* |
| **poly(Lat)2** | **-12.409** | **5.348** | **-2.320** | **0.020** |
| **Lon** | **-0.131** | **0.051** | **-2.548** | **0.011** |
| Pred. index | 0.115 | 0.152 | 0.756 | 0.449 |
| Mass | -0.294 | 0.231 | -1.274 | 0.203 |
| **Hab (alcal)** | **0.526** | **0.112** | **4.703** | **<0.001** |
| ΔpH | 0.066 | 0.066 | 1.003 | 0.316 |
| **NicheNest (dabbl. near shore)** | **-0.929** | **0.275** | **-3.382** | **0.001** |
| NicheNest (dabbl. wetland) | -0.198 | 0.332 | -0.597 | 0.550 |
| **NicheNest (herbiv. wetland)** | **-2.297** | **0.975** | **-2.355** | **0.019** |
| **NicheNest (diving wetland)** | **-1.247** | **0.254** | **-4.903** | **<0.001** |
| **NicheNest (diving cavity)** | **0.626** | **0.314** | **1.995** | **0.046** |
| NicheNest (pisciv. wetland) | -0.088 | 0.246 | -0.357 | 0.721 |
| **NicheNest (pisciv. flexible)** | **-0.545** | **0.263** | **-2.072** | **0.038** |
| **Period** | **0.800** | **0.140** | **5.701** | **<0.001** |
| Pred. index: NicheNest (dabbl. near shore) | -0.169 | 0.158 | -1.072 | 0.284 |
| *Pred. index: NicheNest (dabbl. wetland)* | *0.273* | *0.153* | *1.784* | *0.074* |
| **Pred. index: NicheNest (herbiv. wetland)** | **-1.180** | **0.542** | **-2.177** | **0.030** |
| **Pred. index: NicheNest (diving wetland)** | **-0.488** | **0.136** | **-3.593** | **<0.001** |
| Pred. index: NicheNest (diving cavity) | *-0.126* | *0.070* | *-1.810* | *0.070* |
| Pred. index: NicheNest (pisciv. wetland) | 0.028 | 0.070 | 0.402 | 0.688 |
| **Pred. index: NicheNest (pisciv. flexible)** | **-0.227** | **0.095** | **-2.387** | **0.017** |
| NicheNest (dabbl. near shore): ΔpH | 0.077 | 0.236 | 0.324 | 0.746 |
| NicheNest (dabbl. wetland): ΔpH | 0.205 | 0.322 | 0.638 | 0.524 |
| NicheNest (herbiv. wetland): ΔpH | -0.077 | 0.154 | -0.499 | 0.618 |
| NicheNest (diving wetland): ΔpH | -0.147 | 0.160 | -0.916 | 0.360 |
| NicheNest (diving cavity): ΔpH | 0.024 | 0.096 | 0.246 | 0.806 |
| **NicheNest (pisciv. wetland): ΔpH** | **0.218** | **0.111** | **1.970** | **0.049** |
| NicheNest (pisciv. flexible): ΔpH | -0.094 | 0.166 | -0.565 | 0.572 |
| Period: Pred. index | 0.087 | 0.087 | 0.995 | 0.320 |
| **Period: Mass** | **0.977** | **0.220** | **4.439** | **<0.001** |
| Period: NicheNest (dabbl. near shore) | -0.199 | 0.250 | -0.798 | 0.425 |
| **Period: NicheNest (dabbl. wetland)** | **-0.683** | **0.251** | **-2.719** | **0.007** |
| *Period: NicheNest (herbiv. wetland)* | *2.359* | *1.405* | *1.679* | *0.093* |
| **Period: NicheNest (diving wetland)** | **-1.943** | **0.242** | **-8.039** | **<0.001** |
| Period: NicheNest (diving cavity) | -0.101 | 0.132 | -0.763 | 0.445 |
| **Period: NicheNest (pisciv. wetland)** | -0.631 | 0.145 | -4.360 | **<0.001** |
| Period: NicheNest (pisciv. flexible) | -0.061 | 0.179 | -0.342 | 0.732 |
| **Period: Hab (alcal)** | **-0.499** | **0.125** | **-4.000** | **<0.001** |
| Period: NicheNest (dabbl. near shore): Pred. index | 0.015 | 0.293 | 0.052 | 0.959 |
| Period: NicheNest (dabbl. wetland): Pred. index | 0.118 | 0.300 | 0.394 | 0.693 |
| **Period: NicheNest (herbiv. wetland): Pred. index** | **2.251** | **1.078** | **2.088** | **0.037** |
| **Period: NicheNest (diving wetland): Pred. index** | **-1.078** | **0.262** | **-4.111** | **<0.001** |
| Period: NicheNest (diving cavity): Pred. index | 0.044 | 0.138 | 0.317 | 0.751 |
| Period: NicheNest (pisciv. wetland): Pred. index | 0.050 | 0.138 | 0.361 | 0.718 |
| **Period: NicheNest (pisciv. flexible): Pred. index** | **0.382** | **0.184** | **2.074** | **0.038** |
| Zero-inflation model |  |  |  |  |
| **Intercept** | **-3.244** | **0.707** | **-4.591** | **<0.001** |

Table A8. Parameters for the alternative alien predator and phosphorus model. Intercept is representing *NicheNest* (flexible nesting piscivores) and *Hab* (Oligotrophic). *Pred. index* = predator index, *ΔP* = the change in the phosphorus levels between the study periods. See the variable explanations in the table 2. Dispersion parameter for nbinom2 family = 1.44. Random effect variances: *sp* = 0.07, *SiteID* = 0.38. Significant test values (P < 0.05) are bolded, and trend-setting values (P < 0.1) are shown in italics.

|  | Estimate | SE | z-value | P |
| --- | --- | --- | --- | --- |
| **Intercept** | **-0.720** | **0.227** | **-3.174** | **0.002** |
| poly(Lat)1 | 11.114 | 8.080 | 1.375 | 0.169 |
| *poly(Lat)2* | -10.138 | 5.248 | -1.932 | 0.053 |
| **Lon** | **-0.126** | **0.050** | **-2.527** | **0.011** |
| Pred. index | -0.094 | 0.166 | -0.566 | 0.571 |
| Mass | -0.364 | 0.233 | -1.563 | 0.118 |
| **Hab (eutrophic)** | **0.297** | **0.102** | **2.902** | **0.004** |
| **ΔP** | **0.639** | **0.252** | **2.542** | **0.011** |
| **NicheNest (dabbl. flexible)** | **0.553** | **0.268** | **2.068** | **0.039** |
| NicheNest (dabbl. near shore) | -0.454 | 0.318 | -1.426 | 0.154 |
| NicheNest (dabbl. wetland) | 0.438 | 0.363 | 1.207 | 0.227 |
| *NicheNest (herbiv. wetland)* | *-1.570* | *0.953* | *-1.648* | *0.099* |
| **NicheNest (diving wetland)** | **-0.725** | **0.295** | **-2.455** | **0.014** |
| **NicheNest (diving cavity)** | **1.160** | **0.349** | **3.324** | **0.001** |
| *NicheNest (pisciv. wetland)* | *0.516* | *0.270* | *1.913* | *0.056* |
| **Period** | **0.399** | **0.163** | **2.452** | **0.014** |
| **Pred. index: NicheNest (dabbl. flexible)** | **0.218** | **0.091** | **2.387** | **0.017** |
| Pred. index: NicheNest (dabbl. near shore) | -0.017 | 0.179 | -0.096 | 0.924 |
| **Pred. index: NicheNest (dabbl. wetland)** | **0.466** | **0.161** | **2.889** | **0.004** |
| *Pred. index: NicheNest (herbiv. wetland)* | *-1.036* | *0.552* | *-1.878* | *0.060* |
| *Pred. index: NicheNest (diving wetland)* | *-0.271* | *0.160* | *-1.688* | *0.092* |
| Pred. index: NicheNest (diving cavity) | 0.143 | 0.096 | 1.491 | 0.136 |
| **Pred. index: NicheNest (pisciv. wetland)** | **0.306** | **0.097** | **3.161** | **0.002** |
| **Hab (eutrophic): ΔP** | **-0.561** | **0.170** | **-3.300** | **0.001** |
| NicheNest (dabbl. flexible): ΔP | -0.095 | 0.211 | -0.452 | 0.651 |
| NicheNest (dabbl. near shore): ΔP | 0.092 | 0.261 | 0.354 | 0.723 |
| NicheNest (dabbl. wetland): ΔP | -0.334 | 0.263 | -1.272 | 0.203 |
| NicheNest (herbiv. wetland): ΔP | -0.117 | 0.240 | -0.488 | 0.625 |
| NicheNest (diving wetland): ΔP | -0.135 | 0.251 | -0.536 | 0.592 |
| NicheNest (diving cavity): ΔP | -0.311 | 0.217 | -1.432 | 0.152 |
| NicheNest (pisciv. wetland): ΔP | -0.249 | 0.216 | -1.154 | 0.249 |
| *Period: Pred. index* | *0.270* | *0.154* | *1.751* | *0.080* |
| **Period: Mass** | **1.106** | **0.217** | **5.105** | **<0.001** |
| Period: NicheNest (dabbl. flexible) | 0.040 | 0.177 | 0.224 | 0.823 |
| Period: NicheNest (dabbl. near shore) | -0.349 | 0.304 | -1.150 | 0.250 |
| **Period: NicheNest (dabbl. wetland)** | **-0.679** | **0.275** | **-2.472** | **0.013** |
| Period: NicheNest (herbiv. wetland) | 2.081 | 1.386 | 1.501 | 0.133 |
| **Period: NicheNest (diving wetland)** | **-1.997** | **0.282** | **-7.075** | **<0.001** |
| *Period: NicheNest (diving cavity)* | 0.009 | 0.194 | 0.044 | 0.965 |
| **Period: NicheNest (pisciv. wetland)** | **-0.551** | **0.184** | **-2.993** | **0.003** |
| Period: NicheNest (dabbl. flexible): Pred. index | -0.263 | 0.175 | -1.501 | 0.133 |
| Period: NicheNest (dabbl. near shore): Pred. index | -0.283 | 0.336 | -0.840 | 0.401 |
| Period: NicheNest (dabbl. wetland): Pred. index | -0.046 | 0.309 | -0.149 | 0.881 |
| *Period: NicheNest (herbiv. wetland): Pred. index* | *2.093* | *1.096* | *1.911* | *0.056* |
| **Period: NicheNest (diving wetland): Pred. index** | **-1.343** | **0.308** | **-4.363** | **<0.001** |
| Period: NicheNest (diving cavity): Pred. index | -0.231 | 0.188 | -1.229 | 0.219 |
| Period: NicheNest (pisciv. wetland): Pred. index | -0.170 | 0.188 | -0.906 | 0.365 |
| Zero-inflation model |  |  |  |  |
| **Intercept** | **-3.119** | **0.636** | **-4.903** | **<0.001** |

Table A9. Parameters for the alternative alien predator and water color model. Intercept is representing *NicheNest* (flexible nesting piscivores) and Hab (Oligotrophic). *Pred. index* = predator index, *ΔCol* = the change in the color value between the study periods. See the variable explanations in the table 2. Dispersion parameter for nbinom2 family = 1.70. Random effect variances: *sp* = 0.10, *SiteID* = 0.42. Significant test values (P < 0.05) are bolded, and trend-setting values (P < 0.1) are shown in italics.

|  | Estimate | SE | z-value | P |
| --- | --- | --- | --- | --- |
| **Intercept** | **-0.696** | **0.219** | **-3.174** | **0.002** |
| poly(Lat)1 | 7.445 | 8.100 | 0.919 | 0.358 |
| poly(Lat)2 | -7.824 | 5.148 | -1.520 | 0.129 |
| **Lon** | **-0.123** | **0.051** | **-2.422** | **0.015** |
| Pred. index | -0.155 | 0.169 | -0.915 | 0.360 |
| Mass | -0.346 | 0.225 | -1.534 | 0.125 |
| **Hab (eutrophic)** | **0.335** | **0.107** | **3.121** | **0.002** |
| *ΔCol.* | *0.352* | *0.205* | *1.718* | *0.086* |
| *NicheNest (dabbl. flexible)* | *0.493* | *0.255* | *1.938* | *0.053* |
| NicheNest (dabbl. near shore) | -0.422 | 0.303 | -1.392 | 0.164 |
| NicheNest (dabbl. wetland) | 0.358 | 0.348 | 1.030 | 0.303 |
| NicheNest (herbiv. wetland) | -1.674 | 0.935 | -1.790 | 0.073 |
| **NicheNest (diving wetland)** | **-0.781** | **0.285** | **-2.741** | **0.006** |
| **NicheNest (diving cavity)** | **1.120** | **0.330** | **3.391** | **0.001** |
| *NicheNest (pisciv. wetland)* | *0.464* | *0.257* | *1.803* | *0.071* |
| **Period** | **0.398** | **0.173** | **2.300** | **0.021** |
| **Pred. index: NicheNest (dabbl. flexible)** | **0.259** | **0.093** | **2.790** | **0.005** |
| Pred. index: NicheNest (dabbl. near shore) | 0.110 | 0.177 | 0.623 | 0.533 |
| **Pred. index: NicheNest (dabbl. wetland)** | **0.505** | **0.176** | **2.876** | **0.004** |
| *Pred. index: NicheNest (herbiv. wetland)* | *-1.015* | *0.533* | *-1.904* | *0.057* |
| *Pred. index: NicheNest (diving wetland)* | *-0.270* | *0.157* | *-1.715* | *0.086* |
| Pred. index: NicheNest (diving cavity) | 0.151 | 0.097 | 1.556 | 0.120 |
| **Pred. index: NicheNest (pisciv. wetland)** | **0.353** | **0.099** | **3.558** | **<0.001** |
| **Hab (eutrophic): ΔCol.** | **-0.307** | **0.117** | **-2.632** | **0.008** |
| NicheNest (dabbl. near shore): ΔCol. | -0.068 | 0.203 | -0.337 | 0.736 |
| NicheNest (dabbl. flexible): ΔCol. | -0.196 | 0.246 | -0.798 | 0.425 |
| **NicheNest (dabbl. wetland): ΔCol.** | **-0.525** | **0.254** | **-2.071** | **0.038** |
| NicheNest (herbiv. wetland): ΔCol. | 0.020 | 0.232 | 0.088 | 0.930 |
| NicheNest (diving wetland): ΔCol. | -0.098 | 0.243 | -0.403 | 0.687 |
| NicheNest (diving cavity): ΔCol. | -0.267 | 0.210 | -1.269 | 0.205 |
| **NicheNest (pisciv. wetland): ΔCol.** | **-0.422** | **0.212** | **-1.994** | **0.046** |
| **Period: Pred. index** | **0.360** | **0.156** | **2.305** | **0.021** |
| **Period: Mass** | **1.050** | **0.224** | **4.687** | **<0.001** |
| Period: NicheNest (dabbl. flexible) | -0.012 | 0.188 | -0.065 | 0.949 |
| Period: NicheNest (dabbl. near shore) | -0.237 | 0.302 | -0.784 | 0.433 |
| **Period: NicheNest (dabbl. wetland)** | **-0.766** | **0.292** | **-2.624** | **0.009** |
| Period: NicheNest (herbiv. wetland) | 2.245 | 1.402 | 1.601 | 0.109 |
| **Period: NicheNest (diving wetland)** | **-2.084** | **0.294** | **-7.087** | **<0.001** |
| *Period: NicheNest (diving cavity)* | -0.057 | 0.205 | -0.279 | 0.780 |
| **Period: NicheNest (pisciv. wetland)** | **-0.609** | **0.196** | **-3.109** | **0.002** |
| *Period: NicheNest (dabbl. flexible): Pred. index* | *-0.305* | *0.178* | *-1.712* | *0.087* |
| Period: NicheNest (dabbl. near shore): Pred. index | -0.231 | 0.328 | -0.705 | 0.481 |
| Period: NicheNest (dabbl. wetland): Pred. index | -0.115 | 0.337 | -0.340 | 0.734 |
| *Period: NicheNest (herbiv. wetland): Pred. index* | *1.935* | *1.059* | *1.827* | *0.068* |
| **Period: NicheNest (diving wetland): Pred. index** | **-1.450** | **0.301** | **-4.817** | **<0.001** |
| Period: NicheNest (diving cavity): Pred. index | -0.296 | 0.190 | -1.558 | 0.119 |
| Period: NicheNest (pisciv. wetland): Pred. index | -0.216 | 0.192 | -1.126 | 0.260 |
| Zero-inflation model |  |  |  |  |
| **Intercept** | **-3.153** | **0.729** | **-4.326** | **<0.001** |

Table A10. Parameters for the alternative alien predator and pH model. Intercept is representing *NicheNest* (flexible nesting piscivores) and *Hab* (Acid.). *Pred. index* = predator index, *ΔpH* = the change in the pH between the study periods. See the variable explanations in the table 2. Dispersion parameter for nbinom2 family = 1.41. Random effect variances: *sp* = 0.07, *SiteID* = 0.41. Significant test values (P < 0.05) are bolded, and trend-setting values (P < 0.1) are shown in italics.

|  | Estimate | SE | z-value | P |
| --- | --- | --- | --- | --- |
| **Intercept** | **-0.943** | **0.233** | **-4.048** | **<0.001** |
| *poly(Lat)1* | *16.213* | *8.277* | *1.959* | *0.050* |
| **poly(Lat)2** | **-12.409** | **5.348** | **-2.320** | **0.020** |
| **Lon** | **-0.131** | **0.051** | **-2.548** | **0.011** |
| Pred. index | -0.112 | 0.171 | -0.655 | 0.513 |
| Mass | -0.294 | 0.231 | -1.274 | 0.203 |
| **Hab (alcal)** | **0.526** | **0.112** | **4.703** | **<0.001** |
| ΔpH | -0.028 | 0.158 | -0.175 | 0.861 |
| **NicheNest (dabbl. flexible)** | **0.545** | **0.263** | **2.072** | **0.038** |
| NicheNest (dabbl. near shore) | -0.384 | 0.310 | -1.239 | 0.215 |
| NicheNest (dabbl. wetland) | 0.347 | 0.360 | 0.964 | 0.335 |
| *NicheNest (herbiv. wetland)* | *-1.751* | *0.951* | *-1.842* | *0.065* |
| NicheNest (diving wetland) | -0.702 | 0.290 | -2.416 | 0.016 |
| **NicheNest (diving cavity)** | **1.171** | **0.343** | **3.415** | **0.001** |
| *NicheNest (pisciv. wetland)* | *0.457* | *0.266* | *1.719* | *0.086* |
| **Period** | **0.738** | **0.178** | **4.138** | **<0.001** |
| **Pred. index: NicheNest (dabbl. flexible)** | **0.227** | **0.095** | **2.387** | **0.017** |
| Pred. index: NicheNest (dabbl. near shore) | 0.058 | 0.176 | 0.330 | 0.741 |
| **Pred. index: NicheNest (dabbl. wetland)** | **0.500** | **0.171** | **2.920** | **0.004** |
| *Pred. index: NicheNest (herbiv. wetland)* | *-0.953* | *0.547* | *-1.742* | *0.082* |
| *Pred. index: NicheNest (diving wetland)* | *-0.261* | *0.156* | *-1.671* | *0.095* |
| Pred. index: NicheNest (diving cavity) | 0.101 | 0.100 | 1.010 | 0.313 |
| **Pred. index: NicheNest (pisciv. wetland)** | **0.255** | **0.100** | **2.542** | **0.011** |
| NicheNest (dabbl. flexible): ΔpH | 0.094 | 0.166 | 0.565 | 0.572 |
| NicheNest (dabbl. near shore): ΔpH | 0.171 | 0.277 | 0.615 | 0.538 |
| NicheNest (dabbl. wetland): ΔpH | 0.299 | 0.351 | 0.851 | 0.395 |
| NicheNest (herbiv. wetland): ΔpH | 0.017 | 0.210 | 0.080 | 0.936 |
| NicheNest (diving wetland): ΔpH | -0.053 | 0.214 | -0.246 | 0.806 |
| NicheNest (diving cavity): ΔpH | 0.118 | 0.172 | 0.682 | 0.495 |
| *NicheNest (pisciv. wetland): ΔpH* | *0.312* | *0.182* | *1.719* | *0.086* |
| **Period: Pred. index** | **0.469** | **0.166** | **2.818** | **0.005** |
| **Period: Mass** | **0.977** | **0.220** | **4.439** | **<0.001** |
| Period: NicheNest (dabbl. flexible) | 0.061 | 0.179 | 0.342 | 0.732 |
| Period: NicheNest (dabbl. near shore) | -0.138 | 0.290 | -0.475 | 0.635 |
| **Period: NicheNest (dabbl. wetland)** | **-0.621** | **0.290** | **-2.146** | **0.032** |
| *Period: NicheNest (herbiv. wetland)* | *2.421* | *1.394* | *1.736* | *0.083* |
| **Period: NicheNest (diving wetland)** | **-1.882** | **0.282** | **-6.685** | **<0.001** |
| Period: NicheNest (diving cavity) | -0.040 | 0.196 | -0.202 | 0.840 |
| **Period: NicheNest (pisciv. wetland)** | **-0.570** | **0.189** | **-3.016** | **0.003** |
| **Period: Hab (alcal)** | **-0.499** | **0.125** | **-3.999** | **<0.001** |
| **Period: NicheNest (dabbl. flexible): Pred. index** | **-0.382** | **0.184** | **-2.074** | **0.038** |
| Period: NicheNest (dabbl. near shore): Pred. index | -0.367 | 0.326 | -1.126 | 0.260 |
| Period: NicheNest (dabbl. wetland): Pred. index | -0.264 | 0.331 | -0.795 | 0.426 |
| *Period: NicheNest (herbiv. wetland): Pred. index* | *1.869* | *1.088* | *1.719* | *0.086* |
| **Period: NicheNest (diving wetland): Pred. index** | **-1.460** | **0.298** | **-4.894** | **<0.001** |
| *Period: NicheNest (diving cavity): Pred. index* | *-0.338* | *0.196* | *-1.721* | *0.085* |
| *Period: NicheNest (pisciv. wetland): Pred. index* | *-0.332* | *0.197* | *-1.690* | *0.091* |
| Zero-inflation model |  |  |  |  |
| **Intercept** | **-3.244** | **0.707** | **-4.591** | **<0.001** |

**Online Resource Appendix A Finnish national waterbird surveys and status of the study species**

Two censuses of waterbird surveys were performed using standardized methods between late April and early June starting soon after the ice melts, which is gradually later towards higher latitudes (Koskimies and Väisänen, 1991). Ice break-up affects the timing of migratory waterbird settlement and, hence, sets an exact time frame for the waterbird surveys (Pöysä 1996, 2019, 2022), making it possible to calibrate the phenology between different areas.

The known areas of sites differ greatly (all areas are not reported by the volunteer counters), ranging from small ponds (< 1 ha) to larger lakes or parts of large lakes (max. 1 600 ha). In this study we do not use the survey area as an explanatory variable. Survey sites can represent a whole lake or just a small fraction of a large lake and therefore, a survey site area does not necessarily describe the size or size related ecosystem processes of the surveyed lakes. Since we study the very same lakes in two different time periods, size is not a compulsory variable.

Ten of the studied waterbird species are red-listed in Finland, 1) near threatened species: red-breasted merganser, common merganser, great crested grebe and red-necked grebe, 2) vulnerable species: Eurasian wigeon, northern pintail, 3) endangered species: tufted duck, common coot, 4) critically endangered species: common pochard (Lehikoinen et al. 2019).

**Online Resource Appendix B Water chemistry data**

Water chemistry data were extracted from the Open Data Service of the Finnish Environment Institute (Finnish Environment Institute VESLA, 2020). The sampling and analytical work has mostly been performed by laboratories of the regional water authorities and gathered into one database by the Finnish Environment Institute (controlling the scheme methods and calibration; Finnish Environment Institute and Centres for Economic Development, Transport and the Environment, 2020). Furthermore, we extracted the data according to season and water depth. We only used water measurements conducted in July and August, which is the open water season everywhere in Finland. As proxies for phosphorus, nitrogen, pH and water color we used measurements taken at 1m depth. Phosphorus is the limiting nutrient in freshwater wetlands and is often used as a eutrophication indicator (Aroviita et al., 2019). The variables we selected are also used by the Finnish environmental authorities to analyze the ecological state (P and N) and the anthropogenic pressure of lakes (clarity, color and pH). We expect water chemistry measurements to reflect the state of the lake at a rather large spatial scale. However, this 5 km buffer is an artificial boundary that was limited by the need to obtain enough water measurement data (i.e., a representative sample size for every habitat class), but also to not consider locally irrelevant chemistry measurements (see Holopainen and Lehikoinen, 2021). Water chemistry sites were distributed widely along the latitudinal gradient (Figure A1).

Water chemistry results differ between the habitat classes: compared to oligotrophic and semi-mesotrophic lakes, mesotrophic and eutrophic lakes have a tendency for higher nutrient levels, lower water clarity and darker water colour (Figure A2). On the other hand, pH-levels tend to be lower in oligotrophic and mesotrophic lakes compared to others (Figure A2, see also Holopainen and Lehikoinen, 2021). In this study, we measured the change occurred in the water chemistry levels to find out the effect on waterbirds (Figure A3).

C D


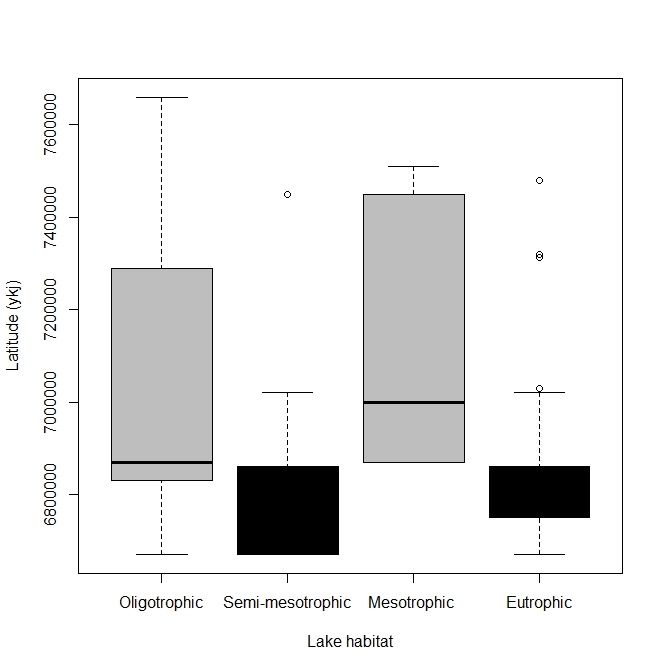

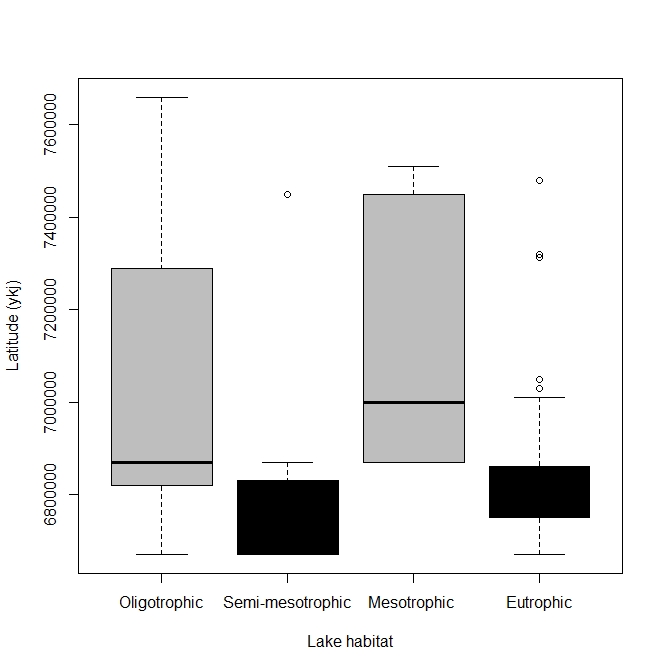

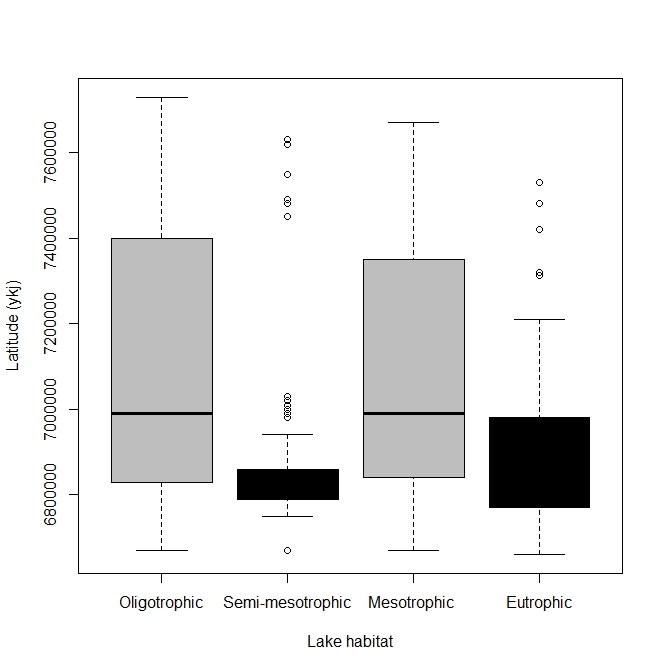

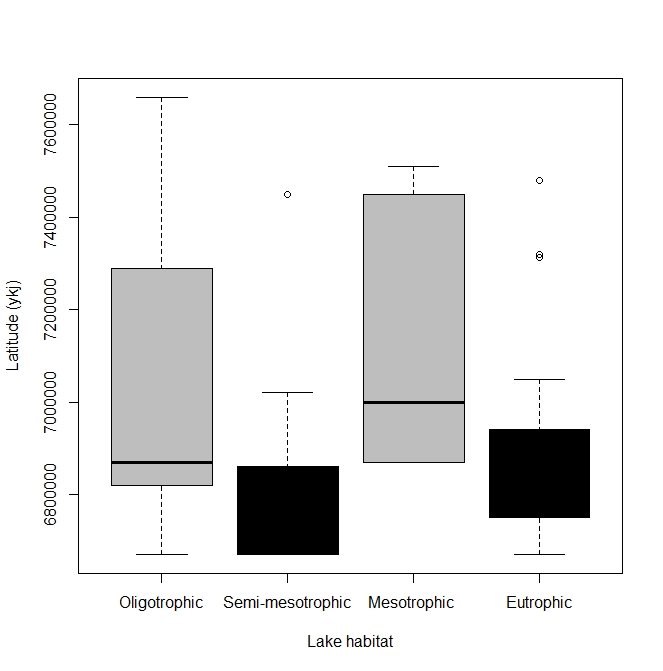
Figure A1. Habitat class-specific latitudes of a) all the waterbird observation sites (N=907), b) total phosphorus sites (N=141), c) water colour sites (N=125) and d) pH sites (N=142). Box plots show the median, interquartile range and whiskers indicate the range. Circles indicate outliers.

A B

**
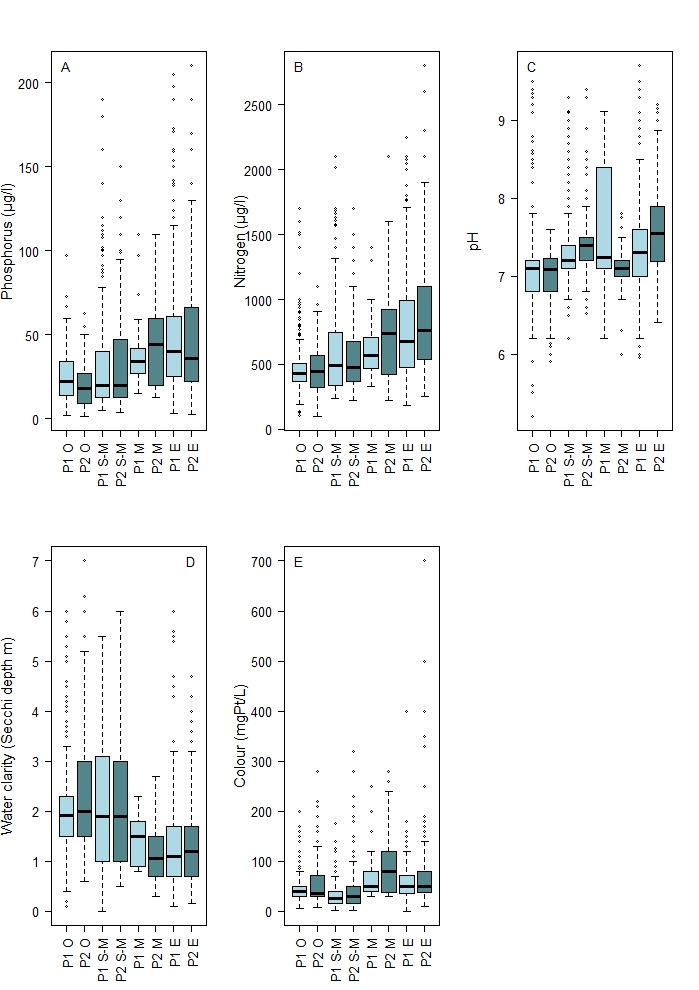
**

Figure A2. Water chemistry measurements of 152 waterbird surveys sites for a) total phosphorus (141 sites), b) total nitrogen (127 sites), c) pH (142 sites), d) water clarity (130 sites) and e) water colour (125 sites) for periods 1 (P1: 1986-1997) and 2 (P2: 20010-2021). O = oligotrophic lakes, S-M = semi-mesotrophic lakes, M = mesotrophic lakes, E = eutrophic lakes. Box plot shows the median, interquartile range and whiskers indicate the range. Circles indicate outliers.


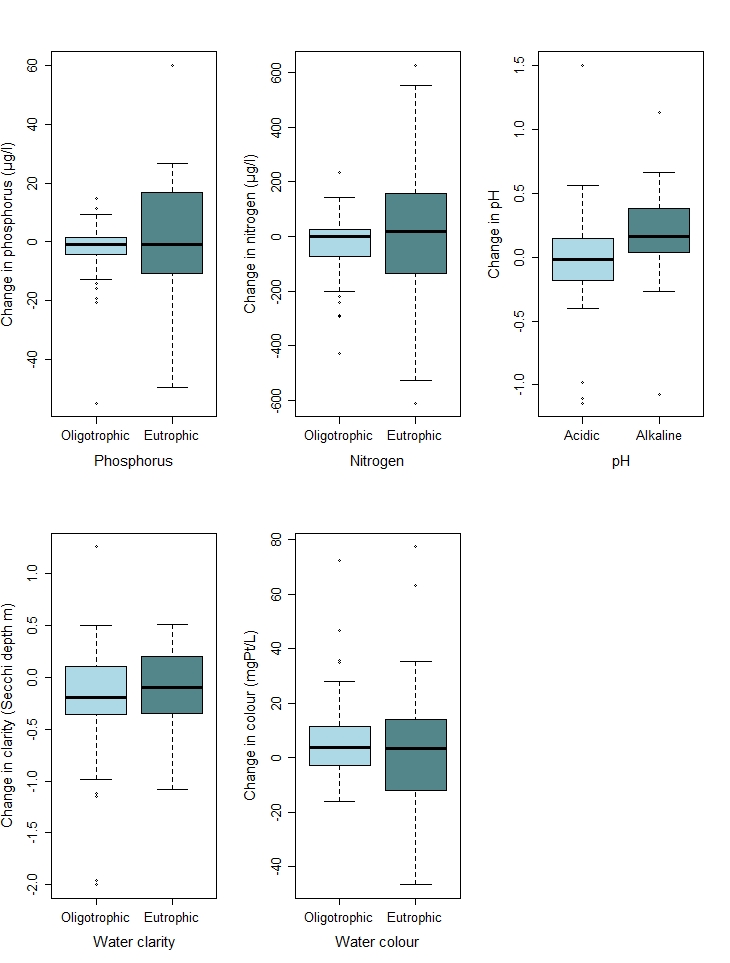


Figure A3. Change in water chemistry from period 1 (1986-1997) to 2 (2010-2021) at 152 waterbird surveys sites for total phosphorus (141 sites), total nitrogen (127 sites), pH (142 sites), water clarity (130 sites) and water colour (125 sites). Light colour indicates oligotrophic or acidic habitat class, dark colour eutrophic or alkaline habitat class. Box plot shows the median, interquartile range and whiskers indicate the range. Circles indicate outliers.

**Online Resource Appendix C** **Alien species population sizes**

Predator densities vary greatly between different European countries (Roos et al., 2002) and this is also the case for invasive alien species in the progress of dispersing into new areas (Kauhala and Kowalczyk, 2011). Currently American mink (*Neovison vison*) inhabit the entire Finland, while the raccoon dog (*Nyctereutes procyonoides*) is still absent from northernmost Lapland (Kauhala 1996a). There are no population estimates for raccoon dogs in Finland, but Finnish hunting bag statistics shows an enormous increase in raccoon dog numbers over time. Harvest bags have been estimated since the season 1976/1977 (min 5 300 in 1977, max 212 500 in 2016; Figure A4). While the estimates of annual numbers killed suffer from several uncertainties (caused e.g. by hunting effort) in their interpretation, these numbers in the bags can be assumed to indicate to some extent the increased population levels in Finland. In our data raccoon dog harvest indices were highest in southern Finland and gradually decreased northwards (i.e. index value variance 0–3.4 from north to south). The data on invasive alien predator abundance consisted of harvest reports from 253 wildlife management associations reporting raccoon dog harvest (see also Figure A5). Trapping index of the raccoon dog in 1986–1989 showed a similar latitudinal pattern, but used a coarser scale (i.e. variance 0–3.1 from north to south; Helle and Kauhala, 1991). While the two indices are not directly comparable, they indicate that relative abundance of the raccoon dog has remained relatively stable. Thus, while raccoon dogs have been increasing, we can assume that the relative density of the species has stayed rather stable between the study periods (i.e. the most raccoon dog abundant areas in the 1980s had highest raccoon dog densities also in the 2020s).


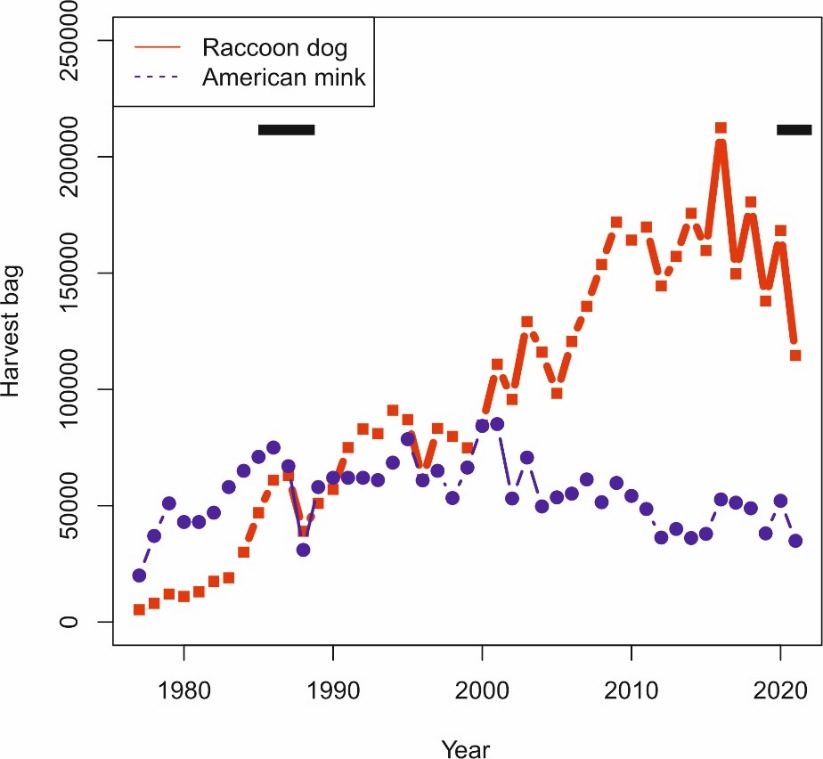


Figure A4. The annual raccoon dog and American mink harvest bag in Finland 1977 – 2021 (Natural Resource Institute Finland, 2021). Black bars indicate waterbird survey years used in this study.

The annual take of American mink has fluctuated (Figure A4). In our data set American mink harvest indices were highest in the archipelago and the lake district in central Finland, decreasing toward the north (274 associations reported mink harvest; see also Figure A6). This pattern was already observed in the 1980s (Kauhala, 1996b). Thus, we again assume rather stable relative densities between the study periods.

In the analysis, we only use the predator indices produced in the years 2014-2021. We assume that while the abundance of the both species have increased during the course of this study, the relative densities of the both species between the areas have remained constant. Our predator index was correlated with latitude (r < –0.6). We, however, wanted to control for the latitudinal effect and since the variance inflation factor value was under 3 when comparing latitude with predator index, we decided to keep latitude in addition to longitude in these models.

We have no reason to believe that the impact of the most important native nest predator species (Holopainen et al. 2020), has increased during the course of the study. In fact, it is more probably decreased. Winter triangle surveys show that the red fox (*Vulpes vulpes*) population has been declining more than 50% since the 1980s, but that the decline has mostly happened in the southern parts of the country, while northern population has slightly increased (Riistakolmiot.fi). Pine marten (*Martes martes)* population has been stable since the 1980s, but again there is some geographical variation: southern population has declined c. 50% while northern has increased c. 80% (Riistakolmiot.fi). Based on the breeding bird surveys population of both hooded crow (*Corvus corone)* and Eurasian magpie (*Pica pica*) have decreased in Finland c. 40 % since 1980s (Lehikoinen & Väisänen, 2023).


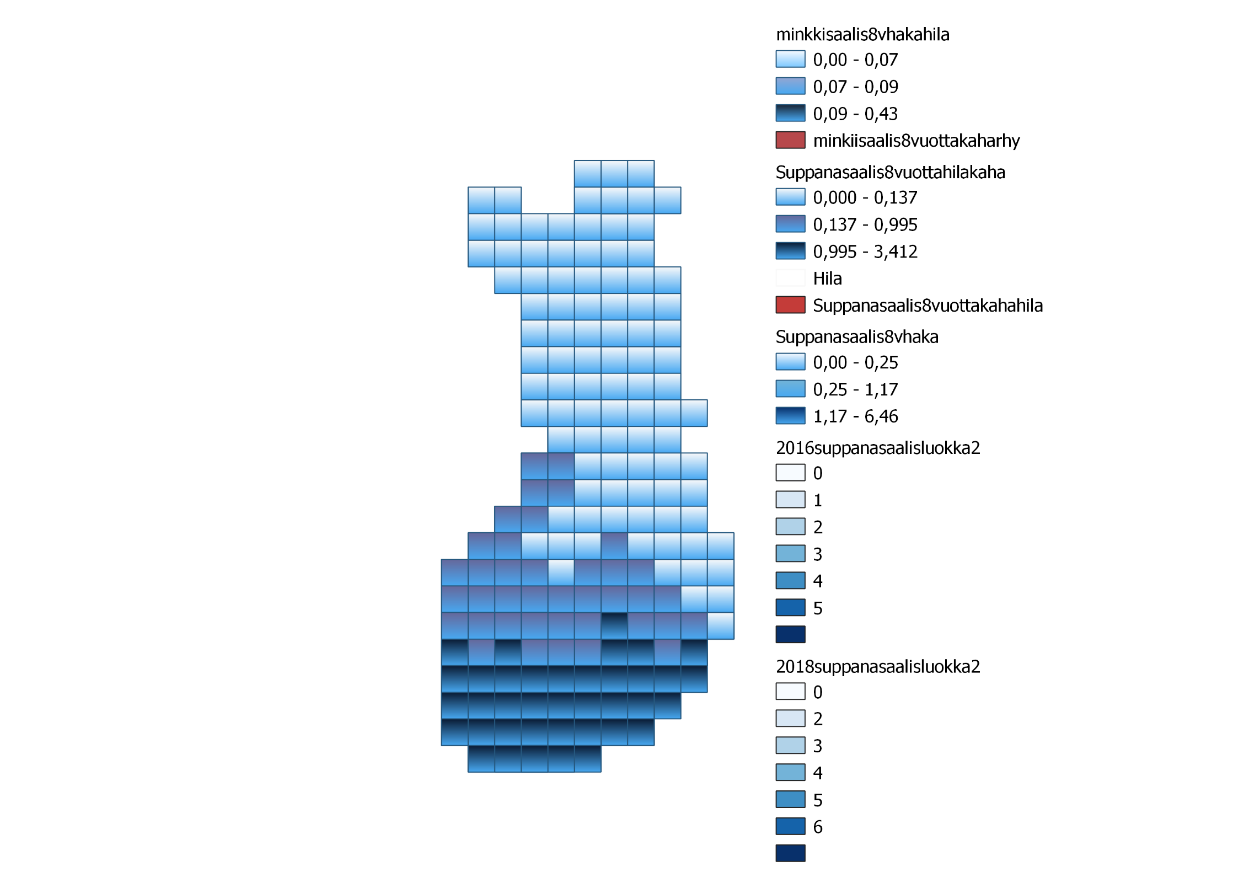


Figure A5. Harvest bag of the raccoon dog in Finland: annual average kills per hectare for each wildlife management unit for the period 2014-2021 averaged for 50x50km grids. Grids are divided in three classes: light blue: 0.0 – 0.137.; blue: 0.137 – 0.995; dark blue 0.995 – 3.412 kills/ha.


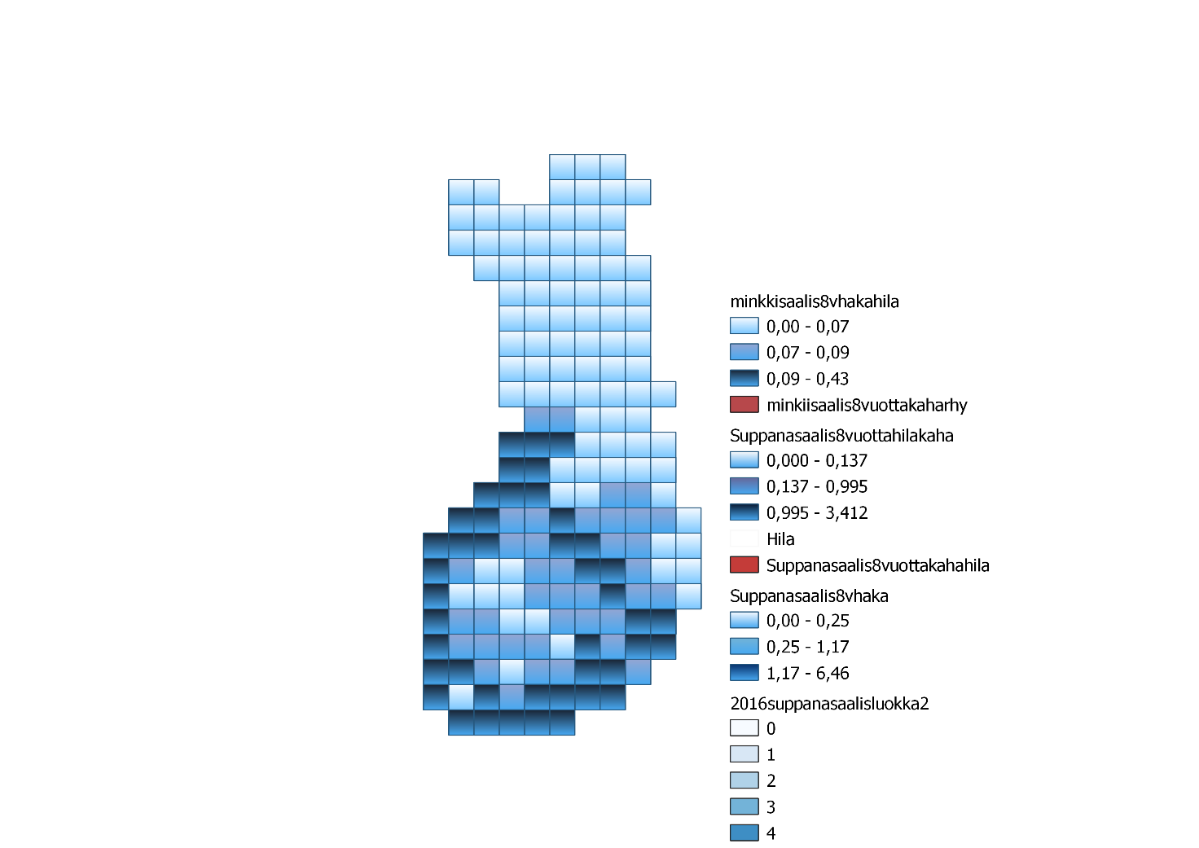


Figure A6. Harvest bag of American mink in Finland: annual average kills per hectare for each wildlife management unit for the period 2014-2021 averaged for 50x50km grids. Grids are divided in three classes: light blue: 0.0 – 0.07; blue: 0.07 – 0.09; dark blue 0.09 – 0.43 kills/ha.

**Online Resource Appendix D The global model (see Table 2 for the explanations).**

Pairs_ijw_ ~ Lat_w_ + Lon_w_ + Predator index_w_ + Mass_j_ + Hab_hw_ + ΔChemistry_w_ + NicheNest_j_ +

Predator index_w_: Mass_j_ + Predator index_w_: Hab_w_* +

Hab_w_: ΔChemistry_w_ + NicheNest_j_: ΔChemistry_w_ +

Period +

Period: Predator index_w_ + Period: Mass_j_ + Period: NicheNest_j_ + Period: Hab_w_ +

Period: Predator index_w_: Mass_j_ + Period: Predator index_w_: Hab_w_ * + Period: Predator index_w_: NicheNest_j_ +

(1|Species_j_) + (1|Wetland ID_w_)

Where i indicates year (1986-1989, 2020-2021), j species and w wetland.

*not included to the pH-model


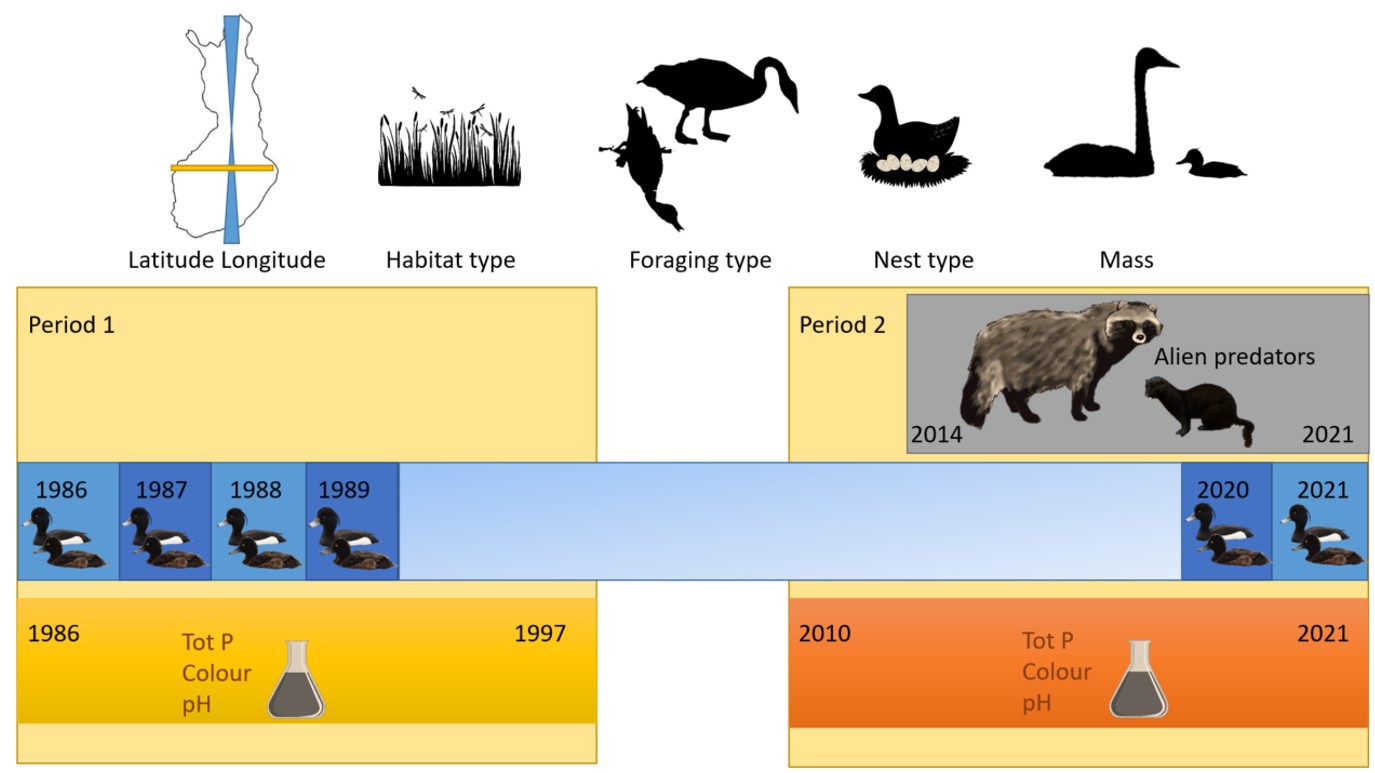


Figure A7. Graphical illustration of the variables used in this study. Constant variables are shown in the upper panel. Waterbird surveys were conducted in 1986–1989 and 2020–2021. Water chemistry data were from years 1986–1997 and 2010–2021 and lake-specifically averaged within periods. Alien predator data were gathered in 2014–2021 and used as constant index for both periods.

**References**

Aroviita, J., Mitikka, S., & Vienonen, S., 2019. Status classification and assessment criteria of surface waters in the third river basin management cycle. In Finnish. *Suomen ympäristökeskuksen raportteja* 37. p. 177. Available at hdl.handle.net/10138/306745.

Cramp, S., Simmons, K., Ferguson-Lees, I., Gillmor, R., Hollom, P., Hudson, R., Nicholson, E., Ogilvie, M., Olney, P., Voous, K., & Wattel, J., 1986. *Handbook of the Birds of Europe the Middle East and North Africa, The Birds of Western Paleartic*. Oxford University Press, Oxford.

Elmberg, J., Arzel, C., Gunnarsson, G., Holopainen, S., Nummi, P., Pöysä, H., & Sjöberg, K. (2020). Population change in breeding boreal waterbirds in a 25‐year perspective: What characterises winners and losers? *Freshw. Biol.* 65, 167–177.

Finnish Environment Institute and Centres for Economic Development, Transport and the Environment, (2020). The status of surface waters, SYKE open web services. Available at https://www.syke.fi/enUS/Open_information/Open_web_services/Environmental_data_API#Hydrology.

Helle, E., & Kauhala, K. 1991. Distribution History and Present Status of the Raccoon Dog in Finland. *Holarctic Ecology*, 14, 278–286.

Holopainen, S., Cehovska, M., Jaatinen, K., Laaksonen, T., Linden, A., Nummi, P., Piha, M., Pöysä, H., Toivanen, T., Väänänen, V.-M., & Lehikoinen, A. 2022. A rapid increase of large-sized waterfowl does not explain the population declines of small-sized waterbird at their breeding sites. *GECCO*, 36, 02144 .

Holopainen, S., Väänänen, V.-M., Fox, & A.D. (2020). Landscape and habitat affect frequency of artificial duck nest predation by native species, but not by an alien predator. Basic Appl. Ecol., 48, 52–60. doi.org/10.1016/j.baae.2020.07.004.

Kauhala, K., 1996a. Introduced carnivores in Europe with special reference to central and northern Europe. *Wildlife Biol*. 2, 197–204.

Kauhala, K. 1996b. Distributional history of the American mink (*Mustela vison*) in Finland with special reference to the trends in otter (*Lutra lutra*) populations. *Ann. Zool. Fennici*, 33, 283–291.

Kauppinen, J., 1993. Densities and habitat distribution of breeding waterfowl in boreal lakes in Finland. *Finnish Game Res*. 48, 24–45.

Koskimies, P., & Väisänen, R.A., 1991. Monitoring Bird Populations. *A Manual of Methods Applied in Finland*. Zoological Museum, Finnish Museum of Natural History.

Lehikoinen, A., Below, A., Jukarainen, A., Laaksonen, T., Lehtiniemi, T., Mikkola­Roos, M., Pessa, J., Rajasärkkä, A., Rusanen, P., Sirkiä, P., Tiainen, J., & Valkama, J., 2019. Breeding population sizes of Finnish birds, in *Linnut­vuosikirja* 2018 (in Finnish with English summary), BirdLife Finland, Helsinki, 38–45.

Lehikoinen, A. & Väisänen, R. A. 2023. Monitoring population changes of land bird species breeding

in Finland in 1975–2022. – Linnut-vuosikirja 2022: 14–29 (in Finnish with English summary).

Natural Resource Institute Finland, 2021. Pienriistasaalis metsästysvuosina 1976/1977 - 1994/1995 ja kalenterivuosina 1996-2021. Available from http://statdb.luke.fi/PXWeb/sq/5c880efe-ede8-45b5-b691-e326c913ade7.

Piha, M., Valkama, J., & Lehikoinen, E., 2018. Suomen lintujen painot ja siipien pituudet – osa 2: ei-varpuslinnut ja varislinnut*. Linnut-vuosikirja*, 166-175. BirdLife Finland (in Finnish with English summary)

Pöysä, H., 1996. Population estimates and the timing of waterfowl censuses. *Ornis Fennica,* 73, 60-68.

Pöysä, H., 2019. Tracking ice phenology by migratory waterbirds: settling phenology and breeding success of species with divergent population trends. *J. Avian Biol*. 50, e02327

Pöysä, H., 2022. Local variation in the timing and advancement of lake ice breaks up and impacts of setting dynamics in a migratory waterbird. *Sci. Total Environ*. 151397.

Riistakolmiot.fi, 2023. Kettu 2023. Available at www.riistakolmiot.fi/report_type/lumijalkilaskennat/. Visited 19.6.2023.

Valkama, J., Vepsäläinen, V., & Lehikoinen, A., 2011. Suomen III Lintuatlas (The 3rd Finnish Bird Atlas). The Finnish Museum of Natural History and the Ministry of the Environment. Available at: http://atlas3.lintuatlas.fi. ISBN 978-952-10-6918-5.
